# The two sides of public debt: Intergenerational altruism and burden shifting

**DOI:** 10.1371/journal.pone.0202963

**Published:** 2018-08-28

**Authors:** Martin Fochmann, Florian Sachs, Abdolkarim Sadrieh, Joachim Weimann

**Affiliations:** 1 Faculty of Management, Economics and Social Sciences, University of Cologne, Cologne, Germany; 2 Faculty of Economics and Management, University of Magdeburg, Magdeburg, Germany; Baylor University, UNITED STATES

## Abstract

In controlled laboratory experiments with and without overlapping generations, we study the role of intergenerational altruism in public debt accumulation. Public debt is chosen by popular vote, pays for public goods, and is repaid with general taxes. We use an optimal control model to derive a theoretical benchmark. With a single generation, public debt is accumulated prudently. With multiple and over-lapping generations, the burden of debt and the risk of over-indebtedness are shifted to future generations. We find a weak but significant sign of intergenerational altruism, observing that the revealed debt preferences increase as the number of following generations decreases. However, we find considerably fewer intergenerational fairness concerns than one would expect on the basis of the behavioral and experimental literature. Instead, political debt cycles that vary with voters’ age emerge. Debt ceiling mechanisms fail to encourage intergenerational altruism and do not mitigate the problem of burden shifting.

## Introduction

Public debt, its accruement, its impact on the economic performance of states, and the question of how over-indebtedness can be successfully avoided are problems that have seized top places on the agenda of economic research for decades. Consequently, the number of theories and approaches that try to deal with these problems is quite high. Pure normative theory tends to look at public debt in a relatively relaxed way. Starting with [1] and [2], the indebtedness of states is modeled as an interplay between a benevolent rational planner, who tries to maximize the welfare of a representative individual with an infinite time horizon, and strictly rational citizens, who adapt their inheritance behavior in order to ensure that their children are able to pay the higher future tax that follows an increase of the public debt.

This rather optimistic normative theory does not, however, fit very well with the empirical observations. The normative model can explain neither the large number of national bankruptcies, nor the huge differences in the indebtedness of different nations. Furthermore, during the last three decades we have observed a sharp increase in the public debt of a couple of important industrial countries that also cannot be easily explained by the normative models of intergenerational altruism and long-term planning. Very recently, this development has caused a dramatic situation in the southern states of the European Union. In the USA, increased public debt has from time to time triggered brief illiquidity episodes for the central government.

Given the high economic impact of public indebtedness, it is essential to understand what the driving forces for the observed phenomena are. This explains the large number of theoretical papers studying the emergence of public debt (see section 2). However, on a behavioral level, little is known about the driving forces and the behavioral motives of an increasing public debt. A better knowledge of the individual motives and preferences would enable democracies to devise measures to deal with worsening public debt.

If we look at public debt from the perspective of individual citizens who vote on taxes and indebtedness, choosing the level of public debt accumulation offers an instrument to adjust the burden of public good provision between current and future generations. The question on the behavioral level is whether people are willing to use this instrument to their own advantage, or whether their *intergenerational altruism* is strong enough to prevent them from exploiting future generations. This is the central research question we deal with in this paper.

The problem is that intergenerational preferences are difficult to observe. The only data we have concern the inheritances people leave for their descendants. But the discussion in the above-mentioned literature has shown that observed inheritance is not closely related to the development of public debt. In our view, controlled laboratory experiments are the most promising way to collect data on the relationship between intergenerational altruism and the willingness to accumulate public debt.

The disadvantage of experiments that mimic a sequence of generations is that their external validity is not guaranteed. We cannot be sure that subjects in an artificial laboratory environment really look at the next experimental “generation” in the same way they look at “real” next generations. Nevertheless, an experimental investigation could be an important first step towards a deeper understanding of intergenerational preferences for the following reason. There is a large body of experimental and theoretical literature on other-regarding preferences (see [3] for a recent overview) that demonstrates that, *within* one generation, preferences of this kind do play a very important role. If experiments with an *intergenerational* design show other-regarding preferences are *not* at work in this context, this could be an important hint that the reason for the strong increase of public debt can at least partly be found in the individual preferences concerning one’s own income and the income of future generations. In this paper, we report the results of an experiment with an intergenerational design and find only very little evidence for other-regarding preferences across generations.

To this end, we introduce a novel experimental design with future generations, public good provision, an income tax system, the risk of over-indebtedness, and debt ceiling mechanisms that enable us to scrutinize many aspects of the issue. An important feature of our design is that it allows us to observe the voting decision on public debt of the whole generation and the revealed debt preference of each single subject at the same time. Thus, we observe not only the aggregated debt on a “macro-level”, but also the “micro-level” public debt decisions. Our design can serve as the nucleus of a new experimental paradigm for research on intergenerational issues in public economics. In section 2, we will discuss our design choice and methodological aspects in more detail. In order to detect altruistic behavior, it is necessary to have a rational, payoff maximizing solution of the decision problem that serves as a benchmark. We use an optimal control model of the debt dynamics to calculate the rational choice solution for rational, payoff maximizing individuals. The optimal control model provides the most straightforward symmetric solution to our dynamic, intergenerational game with a stochastic number of periods and players (in the overlapping-generations setting). Other more involved theoretical approaches that allow for asymmetric solutions may also be of some interest, but they would clearly go far beyond the scope of this study.

We find that the main driving force behind the public debt is the intergenerational transmission of the tax burden. Even in small groups that entertain strong social ties across generations we find a strong tendency to consume beyond the intergenerationally sustainable levels. Within a single generation, *without future generations*, we observe a prudent public debt policy that avoids excessive indebtedness by all means. The behavior in these groups is more prudent than risk-neutral optimal public debt policy, but qualitatively the observed and theoretical dynamics almost perfectly match. *With non-overlapping future generations*, we observe a similar behavior within the lifetime of each generation. New debt is substantially increased only towards the end of the lifetime, leaving high levels of intergenerational debt for the next generations. *With overlapping future generations and a stochastic lifespan*, individuals are neither willing to keep the public debt on a low level, nor willing to reduce public debt voluntarily when the debt level is relatively high. As a consequence, our overlapping generations (OLG) economies often run heavily into public debt early on, leaving the current and the next generations at a high risk of over-indebtedness, public bankruptcy, and penalty taxation.

We implement an absolute debt ceiling mechanism to reduce excessive public borrowing in the intergenerational setting, but find that the negative situation is not improved by this measure. The debt ceiling fails to help because it is simply removed by majority vote whenever it sets a binding constraint on new debt. Finally, with overlapping generations we find clear evidence for political cycles that are driven by the age structure of the electorate. Older individuals typically vote for higher levels of new debt than younger individuals. They also tend to vote for the elimination of debt ceilings more often than the younger ones do.

In our experiment, we observe a weak but significant sign of *intergenerational altruism*. In particular, we find evidence that revealed debt preferences increase as the number of following generations decreases. However, the financial consequences of intergenerational concerns are rather small and cannot prevent economies from burdening future generations heavily. Although we find that individuals behave prudently and fairly within one generation, intergenerational fairness has only little influence on the emergence of public debt. We observe intergenerational fairness concerns and altruism to a much smaller degree than one would expect on the basis of the behavioral and experimental literature. Neither the introduction of a debt ceiling measure, which could serve as a signal against an excessive accumulation of debt, nor the introduction of strong social ties across generations manage to encourage intergenerational fairness concerns.

Although we are cautious not to naively claim full external validity for our experiment, our results are in remarkable accordance with phenomena we observe in reality. Both the sustained increase of public debt that we observe in many developed countries as well as the repeated failure of debt ceilings are reproduced in the laboratory. This encourages us to believe that the experimental approach—in our study and beyond—can help to understand the behavioral foundations of public debt emergence.

The remainder of the paper is organized as follows. In section 2, we provide a brief review of the related literature and discuss some methodological aspects of the experimental design we use. This is necessary because we could not build up on an existing experimental standard design for the investigation of public debt problems, but had to create a new one. In section 2 we explain in detail why we chose this particular design. Section 3 presents the treatments, the theoretical benchmarks, and the experimental protocol. The results of our study and two robustness tests are presented in section 4. In our last section, Section 5, we summarize and discuss our results.

## Related literature and design motivation

### Related literature

There are a large number of theoretical studies modeling the emergence of public debt based on a multitude of various hypotheses. What all these models have in common is that they do not assume the perfect rationality of politicians and citizens. Starting with [4], one of the most prominent assumptions is that voters are myopic insofar as they do not understand that an increase in public debt today leads to higher taxes and/or higher inflation in the future. Rational, self-interested politicians therefore have an incentive to debt finance benefits for their constituencies in return for their political backing. [5] use precisely this argument to justify why governments have a tendency to increase public debt over time. For this line of reasoning also see [6] and [7].

A related approach is modeled by [8] and [9]. In their models, political parties use public debt as a tool if they are in danger of losing the next election. Right-wing parties in power, for example, may have an incentive to increase debt-financed spending on military equipment to reduce the financial leeway of a subsequent left wing government to spend on redistributive measures.

A second approach concentrates on the intergenerational redistributive effects of public debt. [10] and [11] point out that public debt may lead to an intergenerational Pareto improvement (i.e., welfare increase) if it is implemented to share intergenerational risk. The central idea in this literature is that a combination of public debt, social security payments, and taxes can replace the private risk sharing contracts that are not feasible in an intergenerational setting. The positive risk sharing effect may break down when each generation consists of individuals with heterogeneous preferences.

Obviously, public debt and savings enable the current generation to shift financial burdens between generations. [12] and [13] follow this path, pointing out the central role of the conflict between young and old citizens. While the young prefer lower debt and lower pension payment, the old prefer the opposite. [14] show in a dynamic politico-economic model exactly this pattern: “The presence of young voters induces fiscal discipline, that is, low taxes and low debt accumulation” (p. 2785). This implies that the degree of public indebtedness may depend on the age structure of a society. The temptation to shift burdens to future generations may be attenuated in a broad sense by intergenerational altruism or in the narrow sense by a bequest motive. Thus, in a democratic setting in which voters decide on the extent of debt and deficit, this literature shows that individual preferences play a decisive role for the public debt policy implemented.

This is the point at which behavioral theories should enter the stage. It is an important question whether people vote to increase public debt because they intend to exploit subsequent generations or simply because they are myopic. Although behavioral economists have assembled a very impressive number of stylized facts about individual behavior—in particular also about altruism and fairness—to our knowledge the number of behavioral results concerning the motives underlying public indebtedness is very limited. One exception is [15], who conduct an online survey to find out why it is so difficult to reduce an existing public debt. They find that a great majority of the interviewed subjects have a clear preference for a balanced budget and are generally willing to cut public spending. However, when asked about spending cuts in concrete areas (health care, schooling, etc.) most of the interviewees reject proposals to reduce government spending.

At first sight, dynamic common pool resource (CPR) games with multiple generations seem to cover a problem that has some similarities with public debt. For example, [16] investigate the influence of the informational setting on common pool decisions. [17] examine the case of an intergenerational CPR game in which each generation consists of three players making a single extraction decision each. Varying the growth rate of the CPR across treatments, the authors find that extraction rates are above the sustainable level with slow growing resources and below it with fast growing resources. [18] study a dynamic CPR game with a single generation of players that repeatedly make extraction decisions. They find that most subjects make myopic choices instead of considering the resource dynamics. However, there are two fundamental differences to this strand of literature. First, while investment in a common pool is an individual decision, the decision on public debt is not. Second, public debt capacity is a replenishable resource that—in contrast to most of the naturally occurring CPRs—does not grow. These two differences render it impossible to take the experimental work on common pool resources as a model for our own experimental design.

Especially in societies where rising life expectancy and falling birth rates lead to an ageing process, there is a close link between public debt and the social security system. The focus of interest is the relationship between social security, fertility and public debt (see e.g. [19]). [20] show that the best response a society can give to the ageing process is to increase fertility. However, an increase in public debt tends to lead to a decline in fertility ([21]). The ageing process of a society can, however, be the cause of an increasing public debt, because governments could tend to cover the higher social transfers by increasing the public debt ([22], [23]). Although the processes triggered by demographic change could lead to massive intergenerational redistributions, which are both interesting and relevant for analysis, this issue is beyond the scope of our approach.

Because there is obviously no precursor, we have to create a completely novel experimental design. This involves several decisions that can be made in one way or the other. Thus, it seems to be helpful to explain in advance why we decided on the design details as we did.

### Design motivation

We start with a preliminary general methodological remark. The results of experiments are always confronted with the question of their external validity and the answer to this question depends heavily on the experimental design. The likelihood of getting externally valid results increases, the closer the experimental design parallels the real world situation. This implies a fundamental tradeoff between the attempt to create a decision situation in the lab that is as close as possible to reality and the requirement to design experiments in a way that ensures that decisions are simple and easy to control and interpret. A good example is the literature on public good experiments. The public good problem is one of the most important problems that modern societies are confronted with and it is crucial to understand how individuals' decisions are formed and evolve when it comes to the private provision of public goods. The experiments conducted to investigate this question use a standard design that serves to be useful in understanding behavior, even though it deviates in several respects from the public good problems observed in actual settings outside the lab. For example, real public goods typically concern rather large groups. The members of these large groups often have an extremely small effect on the private provision of the public good. In most experiments, however, the effect each individual has on the public good is relatively large because only small groups and rather high marginal per capita returns are implemented. This and other deviations from the real world situation (e.g. the way the public good is actually created) may reduce the external validity of public good experiments, but they are necessary simplifications that allow us to study human behavior in a social dilemma situation.

The same holds for our experiment. In particular, because we are at the very beginning of the research on the behavioral foundations of voting for public debt, we had to find an experimental design that gives first insights in the fundamentals of intergenerational decision-making. Therefore, we decided to build a decision situation that displays a very fundamental choice: *shall I consume public goods at my own expense or shall I shift the burden of financing to someone who succeeds me*? To investigate this question in a controlled and clean setting, we do not include any mechanism that may water down the negative consequences of a shifted burden for the successors, although the incorporation of these elements would possibly increase the external validity of our experiment. There is *no discounting* in our experiment and public debt *does not finance investments* that also benefit future generations. Furthermore, we do not include *interest rates* on debt, *economic growth*, or *income tax progressivity*. Thus, our design does not account for some aspects that are typically associated with public debt, but which are not essential for the core decision described above. An important argument for this research strategy is that the incorporation of one single aspect (like for example an interest rate on public debt) would bias behavior in one direction (less public debt) while the incorporation of another aspect (for example economic growth) would have the opposite effect. Therefore, any selection of realistic features would distract from our research question, which concentrates on the role of intergenerational altruism. Furthermore, by using a design with collective choice and taxation, we omit the intra-generational social dilemma (i.e. the incentives for freeriding that bedevil the voluntary contribution mechanism), focusing only on the intergenerational distribution preferences. Therefore, intergenerational altruism in our experiment is easily identifiable. We observe intergenerational altruism whenever the amount of public debt shifted to the next generation is smaller than the debt burden a group of rational and payoff-maximizing subjects would shift to their successors.

On the other hand, our design accounts for some factors that may be decisive for our core question. In the real world, people voting on public debt do not know when their lives will end. Thus, they play a game with a stochastic ending rule. While most of our treatments deviate from this by using fixed numbers of decision rounds, we account for the “open end” in our overlapping generation treatment, in which subjects do not know exactly how long they are in the game because their “lives” end by a random draw. Our design also accounts for the fact that public debt can *always* be rolled forward except in the case of bankruptcy. Hence, outside bankruptcy no generation is obliged to pay the debt back. Only in the case of bankruptcy, when an economy is over-indebted, do austerity measures requiring strict debt reduction and taxation kick in.

Our design is a compromise between the pursuit of external validity and the necessity to get a clear and unbiased answer to our core question. Given the state of research we are at, it seems to be justified to lay more weight on the second target than on external validity. Nevertheless, our design allows us to investigate the following issues. (1) We compare debt emergence in a non-intergenerational setting with that in an intergenerational setting in order to test if the intergenerational context increases the willingness to accumulate public debt. (2) We compare a non-stochastic lifespan in non-overlapping generations with a stochastic lifespan in overlapping generations. (3) We check the effectiveness of debt ceiling mechanisms. (4) We investigate the influence of a voter’s age on the public debt level and (5) we study the revealed debt preference of each individual in each voting decision.

Finally, a remark on our theoretical model is in order. The purpose of this model is to demonstrate how rational, payoff-maximizing agents vote under the conditions in our experiment. We need this theoretical benchmark to identify altruistic behavior as a deviation from the amount of public debt rational and payoff-maximizing voters would choose.

## Experimental design, theoretical benchmarks and protocol

### The basic experimental design

In all our treatments, an economy exists for 10 or 30 periods. In every period, the economy consists of a group of three individuals. Individuals receive an endowment of 100 cents at the beginning of each period. The group has to make two decisions in every period: the group decides (1) on the size of a public good *PG* and (2) on the mixture of tax and debt with which the public good is financed. In both decisions, the group decides using a median voter mechanism. Individual proposals are not disclosed, but the resulting outcome of the voting, i.e., the median vote, is revealed to the group members.

The public good is a standard linear public good with a marginal per capita return (MPCR) of 0.5, i.e., for every cent invested in the public good, each individual receives 0.5 cents from the public good. In the first decision, each individual chooses a multiple of 30 between 0 and 600 cents (i.e., 0, 30, 60,…, 570, 600 cents) for the public good size. The largest amount that covers at least 2 of 3 votes (i.e., the median vote) is implemented.

After the size of the public good is set, financing is decided by the group. Each group member chooses a tax between 0 and 100 cents (denoted by *τ*_*i*_). The largest amount that covers at least 2 of 3 votes (i.e., the median vote) is implemented. The implemented tax *τ* is automatically collected from every individual’s account, thus making tax evasion impossible. If the total tax revenue is smaller than the size of the public good (i.e., 3*τ* < *PG*), the difference is debt financed. Debt can accumulate over the periods, but it can also be repaid. If the total tax revenue is greater than the size of the public good (i.e., 3*τ* < *PG*), previous debt is first repaid and then savings are accumulated. If the total tax revenue is equal to the public good size (i.e., 3*τ* < *PG*), there is a balanced budget and no debt or savings are accumulated.

After each period, the accumulated debt *D* is checked. If the accumulated debt is not above the safe threshold (*D* ≤ 300), no over-indebtedness and no consequences occur. The safe threshold was chosen to be as large as the total endowment of the three players in a period. This threshold value ensures that the incentives for running into a public debt are high enough but not too high. With it a substantial amount of public good can be provided safely. If the accumulated debt is above the safe threshold (*D* > 300), a random draw decides whether the group is over-indebted and must face consequences. The probability of over-indebtedness increases with the size of the accumulated debt (see Table 1). If over-indebtedness is determined at the end of one period, a tax is imposed in the *following period(s)* until the accumulated debt is reduced to the safe threshold. The imposed tax $\bar{\tau }$ is paid by each group member and, again, no freeriding is possible. If the accumulated debt is greater than 600 cents, the imposed tax equals 100 cents (i.e., the maximum tax level). Otherwise, the imposed tax equals one third of the difference between the accumulated debt and the safe threshold.

**Table 1:** Probability of over-indebtedness.

accumulated debt	probability of over-indebtedness
≤ 300	0%
301 to 400	10%
401 to 500	20%
501 to 600	30%
601 to 700	40%
701 to 800	50%
801 to 900	60%
901 to 1,000	70%
1,001 to 1,100	80%
1,101 to 1,200	90%
> 1,200	100%

$$\bar{\tau }=\{\begin{array}{lll} \frac{D-300}{3} & \text{if} & D\leq 600\\ 100 & \text{if} & D>600 \end{array}$$ (1)

As long as accumulated debt has not been reduced to the safe threshold, no public good can be provided (i.e., *PG* = 0). Note that last period debt is not repaid by the group members. In part of the theoretical literature, it is assumed that citizens have to repay the debt for the last period. We deviate from this assumption because we are interested in the behavior of people who do not have the prospect of having to pay the total debt in the near future.

The payoff *π*_*i*_ of each group member in each period is therefore determined as follows:

$$\pi_{i}=\{\begin{array}{lll} 100-\tau +0.5\cdot PG & \text{if} & \text{no}\;\text{tax}\;\text{is}\;\text{imposed}\\ 100-\bar{\tau } & \text{if} & \text{tax}\;\text{is}\;\text{imposed} \end{array}$$ (2)

When the experiment ends for a subject, the subject is paid the payoff sum from all periods in which the subject was *alive*, i.e., an active member of the economy. Thus, the level of public debt does not directly influence the payoff of a subject. An additional show-up fee is not paid. One cent in the experiment corresponds to exactly one euro cent.

### Treatments

In total, we have six treatments which differ with respect to two dimensions: (1) the variation of the generational configuration (individual lifetime, total lifespan of the economy, generational overlap) and (2) the implementation of an absolute debt ceiling (see Table 2 for a treatment overview). In our two single generation treatments, an economy exists for either 10 (*single-gen 10*) or 30 periods (*single-gen 30*). In both treatments, the economy consists of three group members who remain active in all periods. As there is only a single generation, no intergenerational conflicts can arise. In our multi-generation treatments (*multi-gen*), 3 generations of three subjects participate for 10 periods consecutively without overlap (independent generations). The accumulated debt (or savings) is the only intergenerational connection. In our overlapping generation treatments (*OLG*), each subject “lives” for a stochastic duration. When a subject *dies*, the experiment ends for that subject, who is then paid his/her own “lifetime earnings” (i.e., the accumulated payoff from all active periods). Any subject who dies is replaced by another subject, who starts at the age of 1. The probability of dying increases from period to period. In the first four periods, the probability is 0%. After the fifth period, the probability of dying is 10%, after the sixth period, 20%, and so on until period 14 (100%). Thus, the “lifetime” of a subject in OLG (i.e., number of active periods) is at least 5 and at most 14 periods. As the whole economy starts in the first period, the first generation is not an overlapping generation. Subjects are informed of the general stochastic procedure, but not of the actual birth and death events in their economy. Fig A1 in S1 Appendix shows an example of birth events (i.e., a new subject enters the economy) in each of the generational configurations.

**Table 2:** Treatment overview.

treatment	generations	individual lifetime	total lifespan of economy	debt ceiling	# of independent observations (# of subjects)
single-gen 10	single	10	10	no	6 (18)
single-gen 30		30	30		6 (18)
multi-gen baseline	multiple, independent	10	30	no	8 (72)
multi-gen DC				yes	4 (36)
OLG baseline	multiple, overlapping	stochastic in the interval [5–14]	30	no	6 (74)
OLG DC				yes	6 (75)

With respect to the second dimension, we either implement an absolute debt ceiling mechanism or not. In the baseline treatments, no debt ceiling is applied. In the debt ceiling treatments (DC), public debt is restricted to 300 cents by default. Hence, proposals that imply a public debt level above the debt ceiling are not allowed and technically cannot be submitted. At the beginning of each period, however, the debt ceiling can be removed by majority vote. If the absolute debt ceiling is removed, accumulated public debt can be increased to any level. The debt ceiling can be reinstalled in the following period.

### Theoretic benchmarks

In order to analyze optimal values for the public debt level and public good size, we use optimal control theory as introduced by [24]. We use optimal control theory because it is currently the only approach that enables us to derive theoretical predictions in our rather complicated multi-period, multi-player, overlapping generations game. With our approach we can derive predictions on optimal dynamic choices in our specific framework, but cannot provide a fully fledged game-theoretic analysis of the dynamic game. We will leave these analyses to future research because they go well beyond the scope of this paper.

We assume that the participants want to maximize their expected payoff. Over-indebtedness and individual lifetime in OLG are stochastic variables. ([25] introduced dynamic programming with stochastic variables in optimal control problems.) We assume that the participants choose one of four different actions: (i) pay back as much public debt as possible (*PG* = 0, *τ* = 100), (ii) hold the current public debt level (*PG* = 300, *τ* = 100), (iii) increase the public debt level by a single safe threshold (300) step (*PG* = 600, *τ* = 100), or (iv) increase the public debt level by a double safe threshold (600) step (*PG* = 600, *τ* = 100). We restrict the choices to the points in the action space that lead to critical debt levels, i.e. maximum debt at any given level of default probability. It is evident that increasing debt up to the next critical debt level (i.e. increasing debt without increasing the probability of over-indebtedness) is dominant. Hence, it is sufficient to analyze the dynamic decision at only the critical debt levels. Since we assume zero debt in the *a priori* situation, i.e. birth of the first generation, we can restrict decisions to the steps -300, 0, +300, +600. We label these actions as “-300” or “single step decrease”, “0” or “no change”, “+300” or “single step increase,” and “+600” or “double step increase”. We determine the optimal policy for each treatment and present detailed explanations in S2 Appendix.

**Fig 1:**
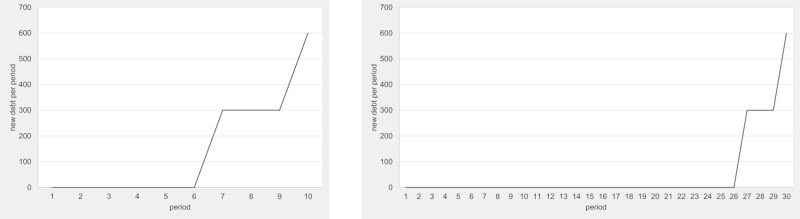
Optimal new debt policy in single-gen 10 and 30 treatments.

**Fig 2:**
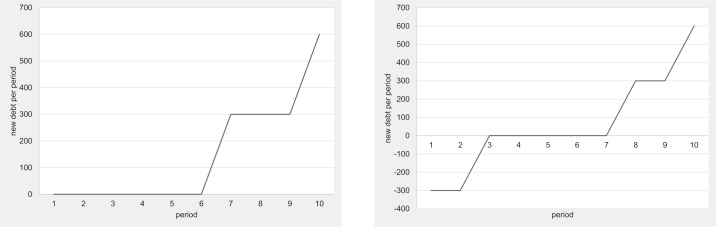
Optimal new debt policy in multi-gen treatments—Starting debt of 0 and 900.

**Fig 3:**
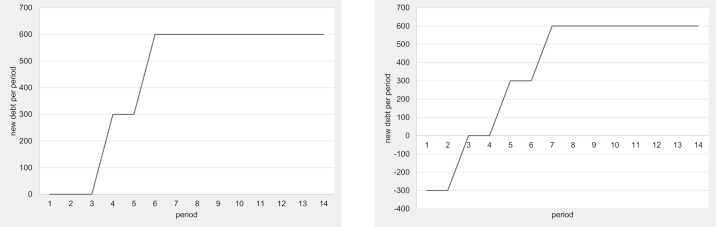
Optimal new debt policy in OLG treatments—Starting debt of 0 and 900.

Figs 1–3 show the optimal new debt policy in our treatments. Note that the displayed optimal new debt can only be implemented in those periods in which the individuals have a choice, but not in a forced debt reduction phase following over-indebtedness. But note also that even after a forced debt reduction phase, the individuals continue with the presented optimal new debt policy of the corresponding period.

The optimal new debt policy in our single-gen treatments (Fig 1) is characterized by a long phase without deficit spending (periods 1–6 in the single-gen 10 treatment and periods 1–26 in the single-gen 30 treatment). Four periods before the termination, public debt is increased. There are three periods with a single step increase, followed by one last period with a double step increase. The optimal new debt policy in the last two periods (9 and 10 or 29 and 30) can only be implemented if forced debt reduction measures are not in place. Hence, the cumulative debt development path is stochastic even if all individuals use the optimal choice policy.

Fig 2 shows the optimal new debt policy in the multi-gen treatments. The solution for the multi-gen treatments is similar to that in single-gen. However, it is expanded by the possibility of different starting points regarding the public debt level. If the starting debt amounts to 0, optimal debt policy is identical to the optimal debt policy in the single-gen 10 treatment. If there is public debt beyond the safe threshold (300), the debt is paid back until it reaches the safe threshold. The only difference between situations with a starting debt and those without is that in the former case the safe part of the debt (up to the safe threshold) is acquired in and retained from the generation before.

Fig 3 shows the optimal new debt policy in the OLG treatments. As before, inherited public debt above the safe threshold is paid back in the early rounds. Unlike the optimal new debt policies in the single-gen and multi-gen treatments, it is optimal in the OLG treatments to increase public debt level earlier on in each individual’s lifetime. While the optimal new debt policy prescribes very high debt levels only towards the end of each individual’s lifetime, the maximum new debt choices come earlier than in the treatments with a fixed life duration. Hence, stochastic life duration has a time discount effect on choices.

It is important to note that in the OLG treatments the three individuals in each economy are typically at different in-game ages. According to our calculations, each individual will vote for the optimal new debt policy that corresponds to her own in-game age. Hence, given our median voter paradigm, the debt policy in each OLG economy is determined by the median aged individual in that economy. This should lead to age-related political cycles in which economies with a young median age vote to acquire less debt than economies with an old median age.

Note that the optimal new debt policy for the treatments with a debt ceiling are identical to those without a debt ceiling because debt ceilings can always be adapted to fit perfectly to the optimal new debt policy.

### Experimental protocol and methods

The experiment was conducted at the University of Magdeburg (MaXLab) and was programmed with z-Tree ([26]). The subjects (mainly students of economics) were recruited with ORSEE ([27]). Written informed consent was obtained by all subjects. Our study did not require an IRB review by German law and the regulation of the German National Science Foundation (Deutsche Forschungsgemeinschaft DFG), because we only use standard experimental protocols. Nevertheless, we provide a statement by an independent expert confirming that our study fully complies with the newly installed set of rules for the ethical conduct of experiments in economics that the German Association of Experimental Economists (Gesellschaft für experimentelle Wirtschaftsforschung GfeW).

Before the experiment started, the subjects were asked to complete a comprehension test to check that they had understood the rules of the game and the math regarding the payoffs. After all the participants successfully finished the comprehension test, they participated in practice runs to familiarize themselves with the game situation. The first practice run consisted of 10 periods of the game with an initial public debt of zero. The second practice run consisted of 5 periods of the game with an initial public debt of 600. Neither the comprehension test nor the practice runs were relevant to the subjects’ payoffs.

In the multi-gen treatments, each generation of subjects was invited to the lab at the same time, with a generous time lag between generations. Subjects were randomly assigned to individual cubicles. After receiving instructions (see S1 File), completing the comprehension test, and participating in the practice runs, the subjects proceeded with the actual experiment. The only difference between the different generations was the size of the public debt at the outset of their in-game lifetime, i.e., the size of public debt that the preceding generations in their economy had accumulated at the time of their in-game birth.

To allow for a smooth transition between our in-game generations in the OLG treatments, we used two separate laboratories located directly next to each other. The subjects for all the generations were invited to one of the two labs at the same time. Each subject was assigned to an individual cubicle. After receiving instructions, completing the comprehension test, and participating in the practice runs, the subjects were randomly assigned to the generations. The first generation subjects were picked up individually and transferred to separate cubicles in the second lab. There they proceeded with the actual experiment. The subjects awaiting their in-game birth remained in the first lab, waiting silently in their cubicles until the lifetime of their in-game predecessor ended. As soon as the predecessor had left the second lab, the successor was transferred to the corresponding cubicle in the second lab, where the “new born” player entered the proceeding OLG economy. The waiting subjects were allowed to read offline, but all forms of communication were prohibited, including e-mailing or texting on mobile phones.

## Results

### The effect of the generational configuration

#### Public debt and public good provision

**Fig 4:**
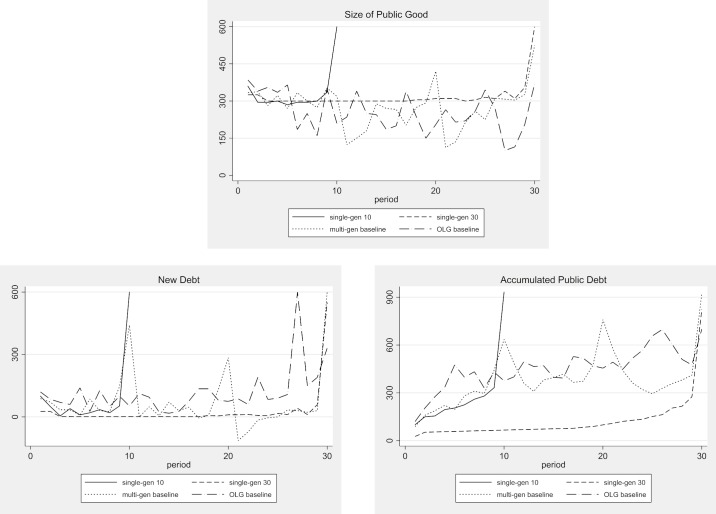
Public good provision, new and accumulated public debt—Baseline treatments.

First, we examine the effect of the generational configuration on the accumulated public debt and on the level of public good provision. Table 3 shows the overall average values of public good provision, of new public debt, and of accumulated public debt in the baseline treatments. The average values for the new public debt exclude the forced debt reduction measure due to over-indebtedness. Fig 4 shows the development of the average values over time. In both single-gen treatments, we observe a moderate increase of public debt over time. Until period 9 in single-gen 10 and period 29 in single-gen 30, respectively, the accumulated public debt on average does not exceed the safe threshold of 300. In these treatments, average public debt rises above 300 only in the last period, in which no risk of over-indebtedness exists. We do not observe over-indebtedness in any of the economies in the single-gen treatments.

**Table 3:** Average observed parameters—Baseline treatments.

treatment		average public good provision	average new public debt	average accumulated public debt	average number of periods with over-indebtedness	average number of periods with imposed tax
single-gen 10		336	93	284	0	0
single-gen 30		318	28	122	0	0
multi-gen baseline		277	73	385	2.63	4.25
OLG baseline		256	103	455	4.67	7.33
**Mann-Whitney U-tests (two-tailed)**				public good provision	new public debt	accumulated public debt
**first 10 periods only**	single-gen 10 vs. single-gen 30			p < 0.001	p < 0.001	p < 0.001
	single-gen 10 vs. multi-gen baseline			p = 0.584	p = 0.408	p = 0.934
	single-gen 10 vs. OLG baseline			p = 0.396	p = 0.056	p < 0.001
**all 30 periods**	single-gen 30 vs. multi-gen baseline			p = 0.330	p < 0.001	p < 0.001
	single-gen 30 vs. OLG baseline			p = 0.010	p < 0.001	p < 0.001
	multi-gen baseline vs. OLG baseline			p = 0.404	p < 0.001	p < 0.001

In contrast to the high degree of prudence that we observe in the economies of our single-gen treatments, all the economies in our multi-gen baseline and OLG baseline treatments quickly accumulate substantial amounts of public debt. While the level of public debt level starts and fluctuates at high levels in both multiple generations treatments, the pattern of debt accumulation and reduction varies over time. In the multi-gen baseline treatment, we observe a strong increase of debt just before and in the periods 10 and 20, i.e., in the last two to three periods of each generation’s lifetime. In the OLG baseline treatment, debt is quickly accumulated from the beginning and is greater than in the multi-gen baseline treatment, except for the periods 9 to 11 and 19 to 21. Comparing the three 30-period treatments, we find significant differences between all three treatments (Mann-Whitney U-test, p < 0.001, two-tailed), with the most debt in the OLG baseline treatment and the least in the single-gen 30 treatment. Comparing the 10-period treatment to the others, we observe a significantly higher (lower) public debt level in the single-gen 10 treatment than in the single-gen 30 treatment (OLG baseline treatment) in the first 10 periods (Mann-Whitney U-test, p < 0.001 for both comparisons, two-tailed). We find no significant difference between the single-gen 10 and the multi-gen baseline treatments. If we compare the public debt levels at the end of an economy, we observe almost the same levels in the single-gen 10, single-gen 30, and multi-gen baseline treatments (22pprox. 900 on average).

All in all, it is remarkable that the behavior in the multi-gen treatment is rather close to the theoretical benchmark. We find signs of intergenerational concern when comparing the end of lifetime (periods 10 and 20) debt levels in the multi-gen baseline to the end-of-lifetime debt levels in the single-gen 10 and 30 treatments. Period 10 in the multi-gen treatment marks the first generation’s end of lifetime, while periods 20 and 30 mark the end of the lifetimes of the second and third generations, respectively. We find the end-of-lifetime debt levels of the first and second generations in the multi-gen treatment are lower than all the end-of-lifetime debt levels of the final generations. This seems to indicate that the non-final generations exhibit some debt restraint when compared to the final generations (i.e., a weak sign of intergenerational fairness concerns). Note, however, that the effect is weak because it is only statistically significant in the comparison of the end-of-lifetime debt level in period 20 of the multi-gen treatment compared to the end-of-lifetime debt level in period 10 of single-gen 10 (Mann-Whitney U-test, p = 0.048, two-tailed). On the aggregated level, we therefore observe only small effects of intergenerational concerns. However, as we will show below, we find much heterogeneity in the subject population’s preference for public debt and we are able to provide significant evidence for intergenerational altruism at the individual level (see Section 4.1.3 on revealed debt preference).

With respect to public good provision, we find that in both single-gen treatments the size of the public good remains almost constant over time except in the two final periods. In contrast, the extent of public good provision varies greatly in the multi-gen baseline and in the OLG baseline treatments. The systematic difference between the single-gen treatments and the multi-gen and OLG treatments is the lack of prudence in the latter. Hence, the economies in the treatments with multiple or overlapping generations are repeatedly over-indebted and are forced to pay imposed taxes. This reduces their overall capability to provide public goods, making them inferior to the single generation economies. In a comparison of the 30-period treatments, the Mann-Whitney U-test picks up a significant difference between the public good provision in the single-gen 30 and the OLG baseline treatments (p = 0.010, two-tailed), but not between the single-gen 30 and multi-gen baseline treatments.

Table 4 confirms the findings so far on a period-by-period basis. There are clearly less deficit periods (about 28%) and more balanced budget periods (about 72%) in the single-gen 30 treatment than in the multi-gen and OLG baseline treatments. Note, however, that individuals with a similar life expectancy (i.e. those in single-gen 10, multi-gen, and OLG) exhibit almost the same frequency of deficit rounds. They differ, however, in the share of balanced budget, voluntary surplus, and imposed austerity rounds. The individuals in the single-gen 10 treatment very rarely accumulate savings and never face imposed austerity measures. In the other two treatments, however, we observe a small fraction of periods (about 11% in the multi-gen baseline and 7% in the OLG baseline) in which the subjects attempt to pay back some of the accumulated debt by voluntarily saving tax income. But, the voluntary debt reduction effort exhibited in these treatments is neither frequent nor sizable enough to offset the accumulated debt. Hence, we observe periods of imposed austerity after over-indebtedness (about 14% in the multi-gen baseline and 24% in the OLG baseline treatments) in which the maximum necessary tax is levied and the full amount used for debt repayments.

**Table 4:** Frequency and average size of observed deficit—Baseline treatments.

treatment	deficit		balanced budget		voluntary surplus		imposed austerity	
	rel. freq	mean	rel. freq	mean	rel. freq	mean	rel. freq	mean
single-gen 10	0.65	146.5	0.33	0	0.02	120	0.00	-
single-gen 30	0.28	97.1	0.72	0	0.00	-	0.00	-
multi-gen baseline	0.49	142.9	0.26	0	0.11	66.9	0.14	233.2
OLG baseline	0.52	158.1	0.17	0	0.07	57.5	0.24	223.0

Note that the relative frequencies are based on the total lifespan of the economies, i.e. 10 periods for the single-gen 10 treatment, but 30 periods for all other treatments.

To address the problem of multiple hypothesis testing, we adjusted our reported p-values in accordance with [28] as a robustness test (see also [29]). In particular, we used the number of comparisons in the respective analysis part (e.g., number of treatment comparisons) and multiply this number with the unadjusted p-value. The adjusted p-value is then compared to the conventional significance level. All our above-described findings are robust to this correction procedure (at least at a 10% level). The only exemption refers to the observed weak sign of intergenerational fairness concerns. The identified statistical significance when comparing the end-of-lifetime debt level in period 20 of the multi-gen treatment with the end-of-lifetime debt level in period 10 of single-gen 10 vanishes. Thus, if the Bonferroni correction procedure is applied, we do not find statistical support for a sign of intergenerational fairness concerns at the aggregated level.

The Tobit regressions presented in Table 5 provide further evidence for the treatment differences discussed above (robust standard errors clustered at the group level are shown in the parentheses under each coefficient). In all the models 1–10 (dependent variable: public debt), only the first 10 periods are considered and the single-gen 10 treatment serves as the reference group. In the b-models, we extend the a-models by adding interaction terms for the treatment dummies and the period.

**Table 5:** Tobit public debt regressions—Baseline treatments.

	model 1-10a (10 periods)	model 1-30a (30 periods)	model 1-10b (10 periods)	model 1-30b (30 periods)
dependent variable	public debt	public debt	public debt	public debt
dummy multi-gen baseline	-3.06	360.76***	52.81	455.26***
	(44.85)	(97.05)	(90.92)	(174.52)
dummy OLG baseline	52.40*	430.66***	223.84***	500.29***
	(27.19)	(94.50)	(66.51)	(172.71)
period	44.89***	12.32***	58.24***	16.13***
	(5.65)	(1.98)	(5.38)	(5.28)
period X			-10.16	-5.79
dummy multi-gen baseline			(12.06)	(5.81)
period X			-31.11***	-4.19
dummy OLG baseline			(7.58)	(5.91)
constant	36.59	-167.70	-36.83	-231.59
	(40.19)	(108.89)	(47.19)	(168.22)
N	200	600	200	600
pseudo R squared	0.0021	0.0496	0.0386	0.0502

In models 1-10a and 1-10b only the first 10 periods are considered and the single-gen 10 treatment serves as the reference group. In models 1-30a and 1-30b, 30 periods are considered and the single-gen 30 treatment serves as the reference group. Robust standard errors are in parentheses.

Test statistics:

*** *p* ≤ 0.01,

** *p* ≤ 0.05,

* *p* ≤ 0.1

We observe no significant effect of the treatment dummy for the multi-gen baseline treatment in any of the models with 10 periods. In contrast, we find a significantly positive effect of the treatment dummy on public debt accumulation for the OLG baseline treatment. Model 1-10b, however, shows that over time debt accumulation in the OLG treatment declines when compared to the single-gen 10 treatment. This is plausible since subjects push their debt accumulation to the end of their lifetime, i.e. towards period 10, in the single-gen treatment. In the OLG treatment, however, debt is accumulated early on and remains on a high level throughout.

In models 1–30, all 30 periods of the respective treatments are considered and the single-gen 30 treatment serves as the reference group. We find a significantly positive overall effect of the treatment dummies for the multi-gen baseline and the OLG baseline treatments on public debt accumulation. While we observe a significantly positive effect of the period, we do not observe any treatment and period interaction effects on public debt.

We run similar Tobit regressions with the public good provision as the dependent variable. The results presented in S3 Appendix (Table C1) support all the findings on public debt.

#### Aggregated payoffs

We use the aggregated payoffs in each economy as a proxy for the generated welfare. Table 6 compares the observed payoffs with the theoretically expected payoffs derived from our optimal control model (see S2 Appendix). The expected payoff in single-gen 30 is higher than in multi-gen baseline and is lowest in the OLG treatment. Hence, the propensity to acquire high levels of public debt early harms the overall intergenerational welfare of the OLG economy. Although the observed payoffs are significantly lower than the expected payoffs in single-gen 30 and multi-gen, the rank order of observed payoffs across all three treatments is the same as that predicted theoretically. Clearly, subjects’ behavior in OLG does not entail enough intergenerational concern to drive total welfare in OLG up and beyond the levels achieved in the other two treatments. In other words, OLG is not only theoretically, but also behaviorally, the environment that induces the highest levels of debt accumulation and the lowest intergenerational welfare.

**Table 6:** Aggregated payoffs.

treatment	observed aggregated payoff of economy	expected aggregated payoff of economy	difference
single-gen 30	14,588	14,826	-238 (p = 0.0273^a^)
multi-gen baseline	14,079	14,652	-573 (p = 0.0173^a^)
OLG baseline	13,545	13,999	-454 (p = 0.1730^a^)
**Mann-Whitney U-Tests, two-tailed**			
single-gen 30 vs. multi-gen baseline	p = 0.028	p = 0.093	
single-gen 30 vs. OLG baseline	p = 0.054	p = 0.199	
multi-gen baseline vs. OLG baseline	p = 0.121	p = 0.897	

^a^ P-value result from Wilcoxon signed-rank test, two-tailed.

As a robustness test, we applied the Bonferroni correction procedure again. The identified significant results when comparing observed and expected aggregated payoffs within one treatment are robust (at least at a 10% level). However, when comparing observed and expected aggregated payoffs between two treatments, only the difference of observed aggregated payoff between single-gen 30 and multi-gen baseline remains statistically significant at a 10% level.

#### Revealed debt preferences

Shifting the focus of our analysis from the performance of the entire economy to the decisions of the individuals, we next analyze the individuals’ preferences for debt-driven public good provision. Remember that the groups first vote and decide on the size of the public good before each individual proposes a tax that will be used to provide the public good. The difference between the suggested tax and the amount needed to pay for the public good is a measure of each individual’s willingness to accept debt-financed public goods. We define the *revealed debt preference* (RDP) as the difference between the budget needed for the provision of the public good (i.e., public good size *PG* chosen by the group) and the total tax revenue that an individual proposes. Formally, our RDP measure can be described as follows:

$$RDP_{i}=PG-3\tau_{i}$$ (3)

where *τ*_*i*_ denotes the (per capita) tax individual *i* proposes. If the proposed tax generates less income than is necessary for the public good provision, our RDP measure will be positive because the individual has revealed a preference for public debt. If, however, the proposed tax generates more income than is necessary for the public good provision, the RDP of the individual will be negative, indicating the individual’s distaste for public debt. An RDP of zero obviously indicates a preference for spending no more on public goods than the taxes generated in the economy.

**Fig 5:**
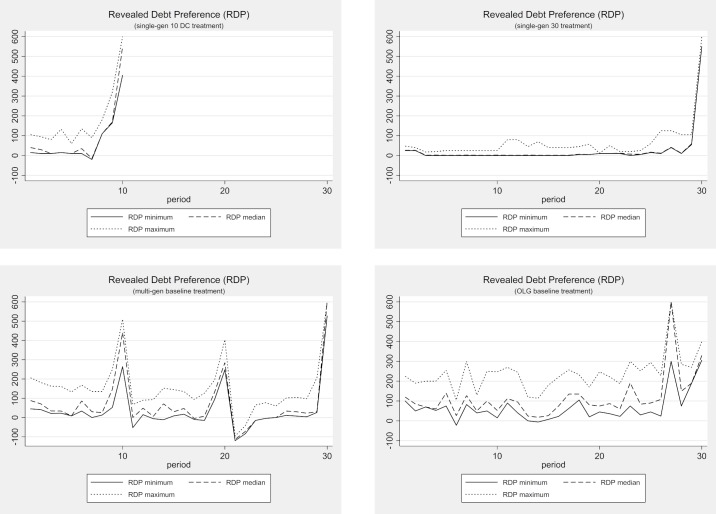
Revealed debt preference (RDP)–Baseline treatments.

Fig 5 displays the development of the RDP in each of the four baseline treatments, including a maximum and a minimum band. In both single-gen treatments, we find very homogeneous revealed debt preferences. The median and the minimum RDP are identical almost throughout the entire lifetime of the individuals and the economy. The maximum RDP runs only slightly above the median and the minimum, indicating that there is very little individual variance in the single-gen treatments. In fact, there is almost no variation at all in the “end-of-lifetime” effect, with almost all subjects voting for the maximum debt.

In the multi-gen baseline treatment, we also observe “end-of-lifetime” peaks, probably because the subjects recognize that they need not fear their own over-indebtedness in the last period of their lives. Note, however, that there is much more variance in these “end-of-lifetime” peaks than we observe in the single-gen treatments. Overall, the individuals in the multi-gen treatment reveal more heterogeneous preferences for debt than those in the single-gen treatments. Knowing that debt creation harms later generations obviously induces heterogeneity in the subject population’s preference for public debt. Some subjects care more about the future generation’s well-being than others. In the OLG treatment, we observe the same type of heterogeneity. Again it seems clear that the individuals have diverse preferences for public debt, most probably due to the heterogeneity of their regard for future generations. Interestingly, the observed maximum RDP in all the treatments seems to be further away from the median RDP than the observed minimum. It seems that on average there are two relatively cautious subjects and one with an extreme preference for public debt accumulation. Nevertheless, even the two relatively cautious individuals vote for debt levels that are not sustainable in the long run.

In fact, comparing the multi-gen treatments’ two end-of-lifetime peaks in which the subjects know that another generation will follow (multi-gen periods 10 and 20) to all other end-of-lifetime peaks (single-gen 10, period 10; single-gen 30, period 30; multi-gen, period 30), we find significantly lower RDPs (Mann-Whitney U-Test, p < 0.02 for all comparisons, two-tailed). These results hint at a small but significant effect of preferences for *intergenerational altruism*. However, since these concerns are not shared by all the individuals, the median voter mechanism generally overrules intergenerational concerns, leading to debt policies that show almost no significant altruism effect at the entire economy level, as we have demonstrated above.

As a robustness test, we applied the Bonferroni correction procedure again. All our above-described findings are robust to this correction procedure (at least at a 10% level). Consequently, at the individual level, we find statistical support for a sign of intergenerational fairness concerns even when the Bonferroni correction procedure is applied.

We wrap up the analysis of the individual behavior with a set of regression models that relate each individual’s revealed debt preference to the individual and environmental parameters. We include elicited risk aversion (Holt-Laury-Task), gender, and an indicator variable that has the value 1 if the individual’s major field of study is in economics or management. Additionally, we include the variables “in-game age”, “lagged debt,” “last generation’s debt,” and “last period imposed tax” in the regression models. Analogous regression analyses relate the individual’s proposed size of the public good and proposed tax to the discussed parameters. All results are presented in Tables C2-C4 in S3 Appendix.

The results of the RDP regression models are straightforward. The revealed debt preference increases with the in-game age in all the treatments. In both single-gen and in the multi-gen treatments, age has a clearly non-linear effect on the RDP. The RDP starts out at a low level, increases rather slowly during most of the individual’s lifetime, and skyrockets towards the end. This is in line with the theoretic benchmark. In the OLG treatment, the non-linear effect of in-game age on the RDP does not show up because in-game life does not end abruptly at a predictable point in time. Instead, the subjects in the OLG treatment gradually and linearly increase their RDP as their in-game age and, thus, their probability of in-game death increases.

Apart from the effect of the in-game age on the RDP, the only other serious effect that we find concerns the last-period-imposed-tax effect in the OLG treatment. Obviously, debt preferences in the OLG treatment jump after each phase of economic austerity (i.e. over-indebtedness and imposed taxes). This suggests that there may be less disciplined action in the OLG treatment than in the others.

Two smaller but also significant effects concern the field of study and the gender. We observe a large negative effect of studying economics in the single-gen 30 treatment, but in no other treatment. Negative effects of studying economics on public good provision are also reported elsewhere, but not in connection with public debt (see e.g. [30]). In the single-gen 10 treatment, we observe a positive effect of males on debt preferences. The effect is only weakly significant and not observed in any other treatment.

The fact that we find no strong reaction of the subjects’ debt preferences to the level of inherited or accumulated debt indicates that they are not backwards looking in their decisions on public debt, as models of direct or indirect reciprocity would assume. (Please note that we observe a small positive effect of lagged debt on debt preferences in the single-gen 30 treatment. The effect is only weakly significant and only present in model 4, but not in model 8 or any of the other treatments. Hence, we have no evidence that this is a robust behavioral effect.)

### The effect of debt ceilings

**Fig 6:**
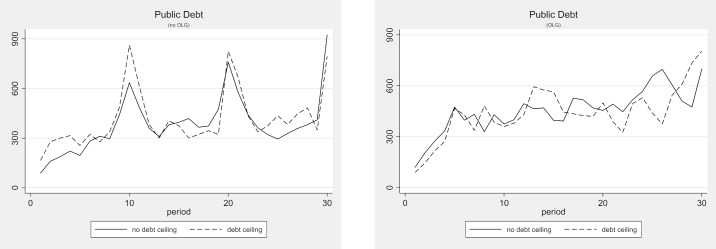
Public debt with and without debt ceiling (multi-gen and OLG).

**Fig 7:**
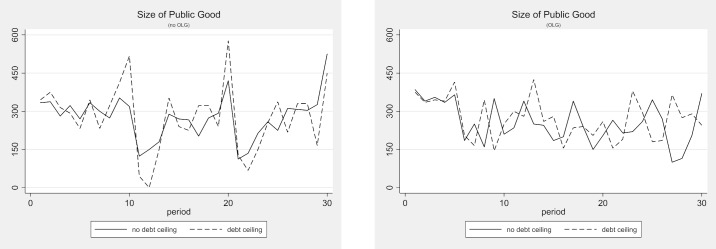
Public good provision with and without debt ceiling (multi-gen and OLG).

In this section, we examine whether introducing a debt ceiling can effectively limit public debt. From a purely game-theoretic perspective, debt ceilings that can be modified at the outset of the period cannot be effective because they are not binding. Table 7 displays the average observed parameters for the multi-gen and OLG treatments with and without a debt ceiling. Figs 6 and 7 depict the development of these parameters over time. These numbers and figures clearly show that introducing a debt ceiling has almost no effect on the provision of public goods and the accumulation of public debt. Using the non-parametric Mann-Whitney U-Test, the only weakly significant difference that we find between the treatments with and without a debt ceiling is a slightly higher size of public debt in the multi-gen treatment with a debt ceiling than without (Mann-Whitney U-test, p = 0.074, two-tailed). This weakly significant difference, however, vanishes if the Bonferroni correction procedure is applied. Hence, we can conclude that debt ceilings do not affect behavior in any economically relevant way. If they make any difference at all, then it seems that they may be worsening the debt situation in the multi-gen setting.

**Table 7:** Average observed parameters—Debt ceiling treatments.

treatment	average public good provision	average public debt	average number of periods with over-indebtedness	average number of periods with imposed tax
multi-gen baseline	277	385	2.63	4.25
multi-gen DC	277	417	4.00	6.25
OLG baseline	256	455	4.67	7.33
OLG DC	271	440	4.50	6.33

**Fig 8:**
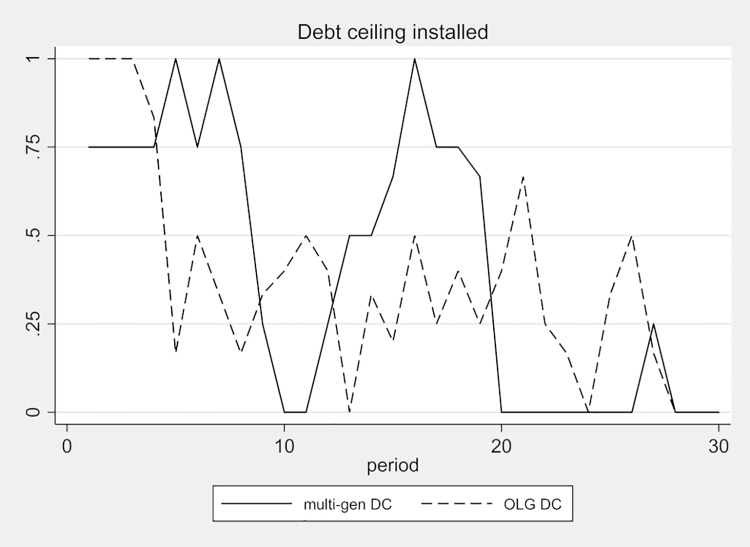
Frequency of debt ceiling installed.

The reason that the debt ceilings are ineffective is that subjects simply lift them basically every time they threaten to constrain new debt. Fig 8 displays the development of the relative frequency of economies with a debt ceiling over time. The striking difference between the multi-gen DC and the OLG DC treatments is that the debt ceilings in the multi-gen DC are all reinstalled after period 11, i.e., after the second generation takes control of the economy. However, the graph in Fig 8 also shows that the attempt to reinstall the debt ceiling is successful only at the beginning, but not sustained. By period 20, the number of multi-generation economies with a debt ceiling is back to zero and the third generation that takes control in period 21 makes no further attempt to reinstall the debt ceiling. We also see attempts to reinstall the debt ceiling in the OLG treatment. But due to the heterogeneous generational mix in the OLG economies, the peaks and troughs of the frequency of installed debt ceilings in the OLG DC treatment do not follow a clear pattern and are less pronounced than in the multi-gen DC treatment.

Table 8 compares the frequencies and average sizes of the observed deficits across the treatments with and without a debt ceiling. While none of the differences are statistically significant, it seems that debt ceilings do reduce the frequency of periods with a deficit and increase the frequency of balanced budgets slightly. But these lower deficit frequencies come at the price of greater average deficits and—in the multi-gen treatment—at the price of almost 50% more periods of imposed austerity, i.e., over-indebtedness.

**Table 8:** Frequency and average size of observed deficit—Debt ceiling treatments.

treatment	deficit		balanced budget		voluntary surplus		imposed austerity	
	rel. freq	mean	rel. freq	mean	rel. freq	mean	rel. freq	mean
multi-gen baseline	0.49	142.9	0.26	0	0.11	66.9	0.14	233.2
multi-gen DC	0.43	189.7	0.32	0	0.05	95.0	0.21	237.0
OLG baseline	0.52	158.1	0.17	0	0.07	57.5	0.24	223.0
OLG DC	0.49	174.8	0.24	0	0.06	132.0	0.21	247.5

As in the previous section, we run Tobit regressions for these treatments with the public debt level and public good size as dependent variables. We use the same model as before, but add the dummy variable “debt ceiling”, which takes the value of 1 if the economy is in a debt ceiling treatment. The debt ceiling dummy exhibits no significant effect on the public debt or the public good in any of the models. The results are displayed in Table C5 in S3 Appendix.

**Fig 9:**
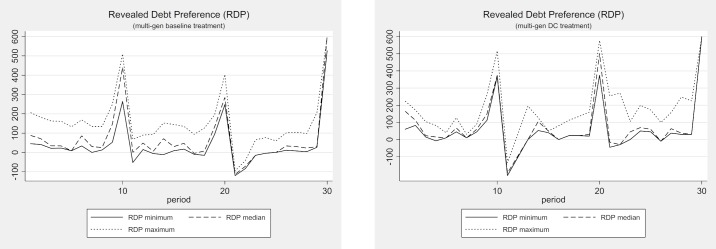
Revealed debt preference (RDP) with and without debt ceiling (multi-gen).

**Fig 10:**
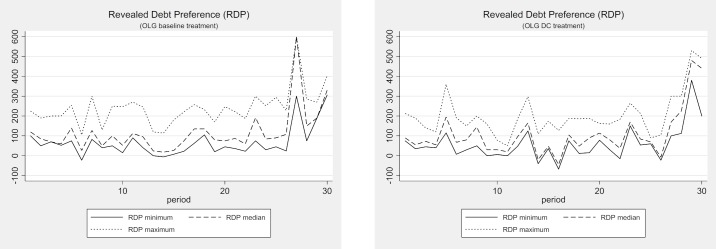
Revealed debt preference (RDP) with and without debt ceiling (OLG).

The development and dispersion of individual revealed debt preferences for the debt ceiling treatments are depicted in Figs 9 and 10. The graphs underline the impression that debt ceilings have no effect on behavior whatsoever. Comparing the development of the revealed debt preferences in the multi-gen treatments with and without debt ceilings, we find the same prominent “end-of-lifetime” peaks. A comparison of the revealed debt preference dispersions reveals that the median and the minimum RDP are very close to each other in both treatments, but the maximum RDP is well above them in almost all periods except in the “end-of-lifetime” periods. The development and dispersion of the revealed debt preferences are also very similar in a comparison of the plots of the OLG treatments with and without debt ceilings in Fig 10.

Please note that we again find weak but significant evidence for intergenerational altruism. As in the multi-gen baseline treatment, the two end-of-lifetime peaks in periods 10 and 20 are significantly lower than the end-of-lifetime peak in period 30 in the multi-gen DC treatment (Mann-Whitney U-Test, p < 0.04 for both comparisons, two-tailed). This effect is robust (at least at a 10% level) when the Bonferroni correction procedure is applied. However, we do not observe any significant positive effect on revealed debt preferences in periods 10 and 20 (compared to the multi-gen baseline treatment), indicating that the debt ceiling measure fails to amplify intergenerational concerns. Similar results are observed in the OLG DC treatment. Most importantly, we do not observe that intergenerational concerns are significantly affected by the debt ceiling.

We also run Tobit regressions that relate the RDP to a debt ceiling dummy and a number of individual and environmental parameters. As can be seen in S3 Appendix (Table C6), they show introducing a debt ceiling has no significant effect on the debt preferences of subjects. Analogous regression analyses relating the individual’s proposed size of the public good and proposed tax to the discussed parameters are presented in Tables C7 and C8 in the S3 Appendix.

### Political cycles

In contrast to the strong “end-of-lifetime” peaks (around the periods 10 and 20) that we observe in the multi-gen treatments, in the OLG treatments we find political cycles driven by in-game age (see, for example, Figs 6 and 7). These political cycles result from the variation in the age structure that the OLG economies undergo due to the random in-game death and birth events. To analyze these political cycles, we divide the members of each economy into two sub-groups. The “seniors” are those individuals who have reached an in-game age of 5 and, thus, face a positive end-of-lifetime probability (i.e., all individuals in the periods 5 to 14). The “juniors” are those individuals who have not yet reached the in-game age of 5 and, thus, are sure to survive at least to the next period (i.e., all individuals in the periods 1 to 4). Table 9 displays the average public good and tax proposals that the juniors and seniors made in the OLG treatment with and without a debt ceiling. In both treatments, the juniors propose significantly less public good provision and significantly higher taxes than the seniors (Mann-Whitney U-test, p ≤ 0.01, two-tailed). Using a Tobit regression on juniors’ revealed debt preferences, we find no significant increase of the debt preferences over time. Hence it seems that juniors do not increase their debt proposals as a response to the higher proposals of the seniors (i.e., social adaptation). They only begin to raise their proposals once they are seniors. Additionally, the number of juniors who vote to keep the current debt ceiling intact (shown in the bottom line of Table 9), is significantly larger than the number of seniors (Fisher Exact Test, p ≤ 0.01, two-tailed).

**Table 9:** Public good and tax proposals.

	OLG baseline			OLG debt ceiling		
	juniors	seniors	test	juniors	seniors	test
Public Good	324	380	p = 0.001	322	380	p < 0.001
Tax	78	58	p < 0.001	80	68	p = 0.010
vote for DC	---	---	---	74%	55%	p = 0.010

Table 10 displays the average new debt per period that results from an economy’s social choice process. We show the averages separately for economies with a majority of juniors and for economies with a majority of seniors. Interestingly, when we compare the individual proposals (see above), the social choice process dilutes the differences between the juniors and the seniors, which are substantial and significant. After the majority vote, we still see a much higher average public debt in the economies with a majority of seniors than in the economies with a majority of juniors (122 to 89 and 141 to 72 in the OLG baseline and OLG DC treatments, respectively). However, the variance in the social choice of the public debt is so large that the Mann-Whitney U-test does not pick up a significant difference in the OLG baseline treatment and only a weak significance in the OLG DC treatment. Additionally, we find that the propensity to install a debt ceiling is significantly higher in the economies with a majority of juniors, being 50% compared to 22% when there is a majority of seniors.

**Table 10:** Voting outcome.

	OLG baseline			OLG debt ceiling		
	more juniors	more seniors	test	more juniors	more seniors	test
new debt	89	122	p = 0.480	72	141	p = 0.061
DC installed	---	---	---	50%	22%	p = 0.001

As a robustness test, we applied the Bonferroni correction procedure again. All our above-described findings are robust to this correction procedure (at least at a 10% level). The only exemption is observed when comparing the average public debt of the economies with more seniors to the economies with more juniors in the OLG DC treatment. In this case, the weak statistical significance vanishes.

### Robustness checks

We complete our analysis by running two robustness checks. To test the robustness of our results with a stricter social choice mechanism, we conducted an additional treatment in which removing and installing the debt ceiling requires unanimity in the popular vote instead of the simple majority. Hence, the debt ceiling in this treatment is only removed (or reinstalled) if all group members agree. From the literature we know that the voting mechanism used in a group decision process may indeed have an influence on the group decisions. [31] show in a common pool resource experiment with groups that groups show more efficient behavior than individuals if they have to use the unanimity rule (compared to majority rule). Surprisingly, we do not observe any significant differences between the decision patterns in the unanimity treatments and our previous majority treatments. All details can be found in S4 Appendix (Appendix D1).

To test whether bequest motives influence behavior when there are social ties across generations, we ran an additional treatment as a robustness check. For this purpose, we used the design of the OLG baseline treatment, but invited groups of friends as our subjects. The only criterion to participate in the experiment as a group of friends is that all individuals must have been friends for at least one year. For each economy, we recruited 3 groups of 5 friends, one for each possible generation. Over time, we replaced each member of the economy whose lifetime ended with one of his or her friends. Note, however, that the three individuals active in an economy were never from the same group of friends. Again, we do not observe any different decision patterns in the OLG treatment with friends as compared to the treatment with strangers. Details can be found in S4 Appendix (Appendix D2).

## Discussion

The aim of this paper is to gain a better understanding of the behavioral foundations and the dynamics of debt emergence. We find that under experimental conditions the main driving force behind the public debt is the intergenerational transmission of the tax burden. Without the possibility to shift the burden of public debt to future generations, individuals vote for a prudent public debt policy that avoids excessive indebtedness. When the burden of debt can be passed to future generations, however, we observe that individuals vote for high levels of public good provision (close to the single generational profit maximization levels) that are debt financed. Over generations massive debt is accumulated, leading to a high risk of financial meltdown followed by penalty taxations. Despite clear evidence of these financial threats for future generations, most individuals are not willing to reduce public expenditure voluntarily. Even in small groups with strong social ties across generations, observed intergenerational fairness effects are too weak when it comes to the accumulation of public debt and the financial consequences of intergenerational concerns are rather negligible.

We additionally study the effect of absolute debt ceilings on the debt financing of public goods. We find debt ceilings that can be modified by popular vote are completely ineffective. The size and speed of debt accumulation is not affected by debt ceilings. Instead, we find that voters quickly learn to adapt debt ceilings to their unabated desire for excessive amounts of debt-financed public goods.

Furthermore, we can show that laboratory economies with overlapping generations create the smooth debt accumulation that is generally observed in real economies. Total debt is higher in the overlapping generation than in the multi-generation economies with homogeneous populations. Additionally, we observe political cycles with overlapping generations. A detailed analysis of these political cycles reveals that public debt problems are exacerbated with aging population as older individuals tend to vote for higher levels of public debt and the elimination of debt ceilings more frequently than younger individuals. Thus, a demographic shift towards a higher aged population is likely to aggravate the financial situation of an economy and to make it more difficult to find a sustainable intergenerational balance in the public budget.

Nearly all of our experimental results show that the observed behavior is very close to the theoretical predictions we derived under the assumption that subjects behave strictly rationally, only maximizing their own monetary payoffs. One important consequence is that the predicted welfare losses caused by over-indebtedness are indeed realized in the experiment. Public debt makes a society worse off if over-indebtedness is a real threat and the inclination to shift burden to the next generation increases public debt and reduces welfare. The most surprising result of our experiments is that we observe fewer intergenerational fairness concerns and less altruism than we had expected based on the behavioral and experimental literature. This literature offers a great number of findings demonstrating that people harbor motivations like fairness, reciprocity, or altruism. However, in the intergenerational context of our experiment, almost all these motivations seem to subside. On the one hand, when confronted with an intragenerational distribution problem, subjects often seem prudent and fair. On the other hand, in the intergenerational distribution decisions in our experiment, subjects behave remarkably payoff maximizing, shifting the heavy burden of public debt to the next generation. Even if the external validity of our experiment is limited, the stark difference between the intragenerational altruism observed in other experiments and the intergenerational altruism *not* observed in our experiment may be an important indicator of the behavioral reasons underlying the excessive public debt observed in many communities and states.

Of course, our experiments do not cover all the particular motives that may attenuate the willingness to bedevil the next generation in reality. The care for their own children may be a much stronger concern for parents than the care amongst friends with strong social ties, which is the setup that we test in our experiment. However, the empirically observed intergenerational transmission of the tax burden of public debt in the last two decades seems to be mirrored in our observations much better than in the predictions of OLG models with more optimistic intergenerational discount rates. Thus, the question to which extent the concern for children really attenuates the problem of increasing public debt remains an open issue for future empirical and experimental research.

## Supporting information

S1 Appendix Appendix A: Birth events. (PDF)

S2 Appendix Appendix B: Optimal control model. (PDF)

S3 Appendix Appendix C: Additional regressions. (PDF)

S4 Appendix Appendix D: Robustness checks. (PDF)

S1 File Instructions of the experiment. (PDF)

S2 File Data. (DTA)
