# Supplementary material for: The two sides of public debt: Intergenerational altruism and burden shifting
Source: PLoS One. 2018 Aug 28;13(8):e0202963. doi: 10.1371/journal.pone.0202963 (PMC6112656; doi:10.1371/journal.pone.0202963)
Supplement: S1 Appendix — (PDF) [file pone.0202963.s001.pdf]

Appendix A: Birth events

Figure A1 shows an example of “birth events” (i.e., a new subject enters the economy) in each generational configuration where the shaded cells indicate “births” in the economy.

single generation (10 periods)

period	cabin 1	cabin 2	cabin 3
1	1	2	3
2	1	2	3
3	1	2	3
4	1	2	3
5	1	2	3
6	1	2	3
7	1	2	3
8	1	2	3
9	1	2	3
10	1	2	3

single generation (30 periods)

period	cabin 1	cabin 2	cabin 3
1	1	2	3
2	1	2	3
3	1	2	3
4	1	2	3
5	1	2	3
6	1	2	3
7	1	2	3
8	1	2	3
9	1	2	3
10	1	2	3
11	1	2	3
12	1	2	3
13	1	2	3
14	1	2	3
15	1	2	3
16	1	2	3
17	1	2	3
18	1	2	3
19	1	2	3
20	1	2	3
21	1	2	3
22	1	2	3
23	1	2	3
24	1	2	3
25	1	2	3
26	1	2	3
27	1	2	3
28	1	2	3
29	1	2	3
30	1	2	3

independent generations

period	cabin 1	cabin 2	cabin 3
1	1	2	3
2	1	2	3
3	1	2	3
4	1	2	3
5	1	2	3
6	1	2	3
7	1	2	3
8	1	2	3
9	1	2	3
10	1	2	3
11	4	5	6
12	4	5	6
13	4	5	6
14	4	5	6
15	4	5	6
16	4	5	6
17	4	5	6
18	4	5	6
19	4	5	6
20	4	5	6
21	7	8	9
22	7	8	9
23	7	8	9
24	7	8	9
25	7	8	9
26	7	8	9
27	7	8	9
28	7	8	9
29	7	8	9
30	7	8	9

overlapping generations

period	cabin 1	cabin 2	cabin 3
1	1	2	3
2	1	2	3
3	1	2	3
4	1	2	3
5	1	2	3
6	1	4	3
7	1	4	3
8	1	4	3
9	1	4	5
10	6	4	5
11	6	4	5
12	6	4	5
13	6	7	5
14	6	7	5
15	6	7	5
16	6	7	5
17	6	7	8
18	6	7	8
19	9	7	8
20	9	7	8
21	9	10	8
22	9	10	8
23	9	10	8
24	9	10	11
25	9	10	11
26	12	10	11
27	12	10	11
28	12	10	11
29	12	10	11
30	12	10	11

Figure A1: Example of birth events in the different generational configurations

Notes: Note that the timing and frequency of births in the single-gen and the multi-gen treatments is always exactly as depicted in the examples in figure A1. In contrast, the example presented for the OLG treatment is just one of the many possible realizations of the stochastic process, in which birth events in OLG immediately follow death events that occur at random between the 5th and 14th “in-game age” of each subject. Notice that each economy runs through its own realization of this stochastic process in our OLG setting.
