# Supplementary material for: The two sides of public debt: Intergenerational altruism and burden shifting
Source: PLoS One. 2018 Aug 28;13(8):e0202963. doi: 10.1371/journal.pone.0202963 (PMC6112656; doi:10.1371/journal.pone.0202963)
Supplement: S2 Appendix — (PDF) [file pone.0202963.s002.pdf]

Appendix B: Optimal control model

Model assumptions

We make the following simplifying assumptions to solve the optimal control problem:

- The individuals aim to maximize their expected payoff.
- The individuals make use of a restricted decision space. The actions are (including the corresponding choices of public good size and tax): (i) pay back public debt as much as possible ($PG = 0, \tau = 100$), (ii) hold current public debt level ($PG = 300, \tau = 100$), (iii) increase public debt level by a single safe threshold (300) step ($PG = 600, \tau = 100$) and increase public debt level by a double safe threshold (600) step ($PG = 600, \tau = 0$). We label these actions as "-300", "0", "+300", and "+600".
- If a participant is indifferent between these options, the decreasing priority should be given by 0, +300, -300, +600.
- We optimize the choice of each individual as if that individual determines the policy for the entire economy. Since actual social choice involves a median voter paradigm, each individual maximizes payoffs by voting for the optimal policy we determine.
- In the OLG treatments the stochastic lifetime of each individual is replaced by a common lifetime because different probabilities of dying would imply different optimal policies. This includes the realization of the stochastic variable.

Only the following changes in public debt level are possible: $-300, 0, 300, 600$. So only the public debt levels $0, 300, \dots, 1800$ can be reached as the economy starts without debt.

Stochastic variables

Let $(\omega_t)_{t\in\{0,1,\dots,N\}}$ be a family of stochastic variables. Further, let $(X_D)_{D\in\{0,300,\dots,1800\}}$ be a family of stochastic variables. $P(X_D = 1)$ represents the probability of over-indebtedness depending on the accumulated debt D as listed in Table 1. The distribution of (ω_t) depends on the state x_t as follows:

$$P\,(\,\omega_t = 1|x_t) := x_2(t) + \left(1 - x_2(t)\right) \cdot \; P\left(X_{x_1(t)} = 1\right)$$

$$= \begin{cases} 0 & \text{for} \quad x_1(t) \leq 300 \\ \dfrac{x_1(t) - 300}{1000} & \text{for} \quad 300 < x_1(t) \leq 1200 \;\wedge x_2(t) = 0 \\ 1 & \text{for} \quad (\,300 < x_1(t) \leq 1200 \;\wedge x_2(t) = 1) \vee x_1(t) > 1200 \end{cases}$$

and $P(\omega_t = 0|x_t) = 1 - P\,(\,\omega_t = 1|x_t)$.

The realization $\omega_t = 1$ means that the economy is over-indebted in period t. Hence, $\omega_t = 0$ stands for an economy without over-indebtedness.

Let $(\nu_t)_{t\in\{\,0,1,\dots,N\,\}}$ be a familiy of stochastic variables. $P(\nu_t = 1)$ is the possibility of life termination in period t. The lifetime in single-gen and multi-gen treatments is a degenerate distribution, as there is no random influence:

$$P(\nu_t = 1) = \begin{cases} 1 & \text{if} \quad t = N - 1 \\ 0 & \text{else} \end{cases} \qquad \forall t \in \{0,1,\dots,N-1\}$$

In the OLG treatments ν_t is calculated as a conditional probability. $P(\nu_t = 1)$ is calculated as the sum of the probabilities of the following events: 1) The individual was alive in period $t-1$ and died in period t and 2) the individual was already dead in period $t-1$.

Figure B1 shows the probability of dying for each period in the OLG treatments.

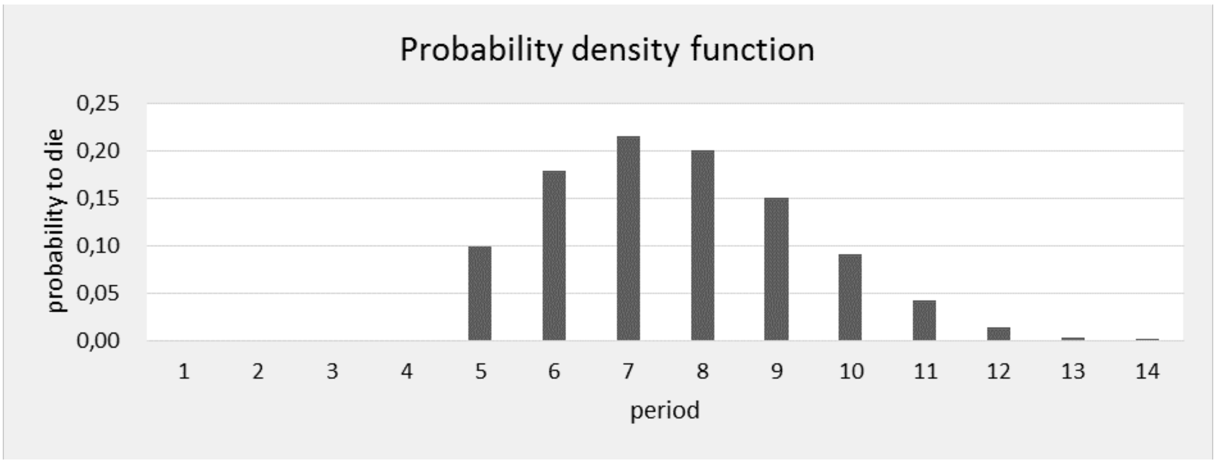

Figure B1: Probability density function of stochastic individual lifetime

Model of optimal control

The state vector $x(t)$ contains three components

$$x_t := x(t) := \begin{pmatrix} x_1(t) \\ x_2(t) \\ x_3(t) \end{pmatrix} \quad \forall t \in \{0,1,\ldots,N\},$$

where N is the maximum number of periods an economy exists. Thus, $N \in \{10, 14, 30\}$.

The first component is the public debt level. The second component is a truth-value, which describes if an over-indebtedness of the last period is still active for the current period. The third component also saves a truth-value. It describes whether a public debt level over 300 was reached without over-indebtedness.

As mentioned, there are four different values for the control variable in period t, which is defined as $u_t \in Y := \{-300, 0, +300, +600\}$.

The states are calculated via a function F, which is defined as

$$x_{t+1} = F(x_t, u_t, \omega_t) \quad \forall t \in \{0,1,\ldots,N-1\}$$

and in detail

$$\begin{aligned} x_1(t+1) &= x_1(t) + (1-\omega_t)\cdot u_t - \omega_t \cdot 300, \\ x_2(t+1) &= \mathbb{I}_{\{a>300\}}\big(x_1(t+1)\big)\cdot \omega_t, \\ x_3(t+1) &= x_3(t) + \big(1-x_3(t)\big)\cdot\big(1-x_2(t+1)\big)\cdot \mathbb{I}_{\{a>300\}}\big(x_1(t+1)\big). \end{aligned}$$

Note that $\mathbb{I}_{\{a>300\}}$ stands for the indicator function, which equals one if the condition in brackets is met by the argument of the function and zero otherwise.

Explanation of the calculations for new states:

1) The new public debt level is calculated as the sum of the last public debt level $x_1(t)$ and either the control variable u_t if there is no over-indebtedness ($\omega_t = 1$) or -300 otherwise.
2) The over-indebtedness of the economy stays active, if the current states show over-indebtedness ($\omega_t = 1$) and the new debt level is greater than 300.
3) The third component shall save the value 1 once this value was reached (ensured by first addend). The second addend contains three factors with values within $\{0,1\}$. It equals 1, when all factors equals 1, i.e. $x_3(t) = 0$, there is no over-indebtedness in period $t+1$ ($x_2(t+1) = 0$) and the new public debt level is greater than 300.

All together, we can define the state space as

$$X \coloneqq (\{0,300\} \times \{0\} \cup \{600,900,1200,1500,1800\} \times \{0,1\}) \times \{0,1\}.$$

In general, the payoff π is the sum of the Mayer term and the Lagrange term with corresponding functions $E(\cdot)$ and $L(\cdot)$:

$$\pi := E(x_0, x_N) + \sum_{t=0}^{N-1} L(x_t, u_t, \omega_t, \nu_t) \quad \text{with } x_N, x_t \in X \text{ and } u_t \in Y \quad \forall t \in \{0,1,\dots,N-1\}.$$

Because of the experimental design, only the decisions u_t are payoff relevant, so $E(x_0, x_N) \equiv 0$. The Lagrange function is defined as follows:

$$L(x_t, u_t, \omega_t, \nu_t) \coloneqq \begin{cases} L(x_t, u_t, \omega_t) & \text{if} \quad \nu_t = 0 \\ 0 & \text{else} \end{cases}$$

$$L(x_t, u_t, \omega_t) \coloneqq \begin{cases} -\infty & \text{if} \quad x_t \notin X \\ -0.01 & \text{if} \quad u_t \neq -1 \quad \wedge \ \omega_t = 1 \wedge x_3(t) = 0 \\ -0.01 & \text{if} \quad u_t \neq 2 \quad \wedge \ \omega_t = 1 \wedge x_3(t) = 1 \\ 0 & \text{if} \quad u_t = -1 \quad \vee (\omega_t = 1 \wedge x_3(t) = 1) \\ 450 & \text{if} \quad u_t = 0 \quad \wedge \ \omega_t \neq 1 \\ 900 & \text{if} \quad u_t = 1 \quad \wedge \ \omega_t \neq 1 \\ 1200 & \text{if} \quad u_t = 2 \quad \wedge \ \omega_t \neq 1 \\ -\infty & \text{else} \end{cases}$$

The function values of the Lagrange function $L(x_t, u_t, \omega_t)$ can be achieved as follows (numbering follows function definition):

1) Infeasible states are penalized, so that they will not be assumed as a value.

2-3) In the case of over-indebtedness, different values for $x_3(t)$ imply different controls. This is done with a slight penalization, which is only active in the case of indifference.

2) If there is over-indebtedness, the public debt is paid back independently of the control variable. The penalization shall only influence the chosen control in the case of indifference for a consistent result. It is clear that this does not affect the payoff.

3) In the case of over-indebtedness and a prior possibility of different trajectories, the control must be set to 2. After the debt has been paid back, the optimal policy at a debt level of 300 in OLG is 2. Hence, it is evident that this has to be considered when choosing the optimal policy.

4-7) Without over-indebtedness only the value of the control variable influences the payoff. Because of the cooperative game, the payoff is tripled in comparison with the individual payoff.

8) For the well-definedness of the function, not provided states are penalized.

With these definitions the stochastic optimal control problem can be formulated as

$$\begin{aligned} &\max_{x,u,\omega,\nu} \mathbb{E}_\nu\left(\mathbb{E}_\omega\left(\sum_{t=0}^{N-1} L(x_t,u_t,\omega_t,\nu_t\right)\right) \\ &\text{s.t.} \quad x_0 = \overline{x_0} \in X \\ &\qquad x_{t+1} = F(x_t,u_t,\omega_t) \qquad \forall t \in \{0,1,\dots,N-1\} \\ &\qquad u_t \in Y \qquad \forall t \in \{0,1,\dots,N-1\} \end{aligned}$$

Bellman (1954) formulated the principle of optimality: “An optimal policy has the property that whatever the initial state and initial decision are, the remaining decisions must constitute an optimal policy with regard to the state resulting from the first decision.” This is the basis of dynamic programming, which finds the solution as a composition of sub-solutions.

Bertsekas (1995) defined the cost-to-go-function for $t \in \{0,1,\dots,N-1\}$ as

$$\begin{aligned} J^*(x_t,t) = &\max_{\substack{x_t,\dots,x_{N-1}\\ u_t,\dots,u_{N-1}}} \mathbb{E}_{\nu_t,\dots,\nu_{N-1}}\left(\mathbb{E}_{\omega_t,\dots,\omega_{N-1}}\left(\sum_{i=t}^{N-1} L(x_i,u_i,\omega_i,\nu_i\right)\right) \\ &s.t. \quad x_{i+1} = F(x_i,u_i,\omega_i) \quad \forall i \in \{t,\dots,N-1\} \\ &\qquad u_i \in Y \qquad \forall i \in \{t,\dots,N-1\} \end{aligned}$$

and $J^*(x_N,N) = 0$. $J^*(\overline{x_0},0)$ equals the maximized expected value of the optimal control problem.

Bertsekas (1995) also proved the following result concerning the algorithmic solution via the principle of optimality. Let $J: X \times \mathbb{N} \to \mathbb{R}$ be a function with $J(x_N,N) \coloneqq E(x_N) \equiv 0$ and for $t = N-1,\dots,1,0$

$$J(x_t,t) \coloneqq \max_{u_t \in Y} \mathbb{E}_{\nu_t}\left(\mathbb{E}_{\omega_t}\big(L(x_t,u_t,\omega_t,\nu_t) + J(F(x_t,u_t,\omega_t),t+1)\big)\right).$$

Then $J(\overline{x_0},0) = J^*(\overline{x_0},0), \forall \overline{x_0} \in X$ and accordingly the resulting (x_t,u_t) are optimal for the optimal control problem.

Algorithmic solution

It can be shown that the function $F(x,u,\omega)$ returns feasible states $z \in X$ if the input data is feasible (i.e. $x \in X, u \in Y, \omega \in \{0,1\}$) and $0 \le z_1 \le 1800$ is true for the first component. So the only check for feasibility is $0 \le z_1 \le 1800$. Furthermore, it can be shown that all resulting states are discrete and elements of the following sets and thus need no discretization.

Every state contains $n_x = 3$ components. There are $N_x = 24$ discrete states. The model contains $n_u = 1$ control variable, which can attain $N_u = 4$ values. We have $N+1$ time points. The

algorithmic implementation needs the following discrete enumerations of the known sets. The prioritization of the control variable influences the order of $\tilde{Y}$.

$$\text{State space: } \tilde{X} = \{x^{(1)}, x^{(2)}, \dots, x^{(N_x)}\}$$
$$= \{(0,0,0)^T, (0,0,1)^T, (300,0,0)^T, (300,0,1)^T, (600,0,0)^T, (600,1,0)^T, (600,0,1)^T, (600,1,1)^T, (1800,0,0)^T, (1800,1,0)^T, (1800,0,1)^T, (1800,1,1)^T\}$$
$$\text{Control space: } \tilde{Y} = \{u^{(1)}, u^{(2)}, \dots, u^{(N_u)}\} = \{2, -1, 1, 0\}$$
$$\text{Time: } \tilde{T} = \{t^{(0)}, t^{(1)}, \dots, t^{(N)}\} = \{0, 1, \dots, N\}$$

We define five characteristic values to determine the quality of an optimal policy:

1) π_0 is the payoff, which can be generated if there shall be no risk, i.e. no positive probability of over-indebtedness.
2) π_μ is the expected payoff under the optimal policy.
3) π_σ is the standard deviation of the payoff under the optimal policy.
4) π_{min} is the minimal possible payoff under the optimal policy.
5) π_{max} is the maximal possible payoff under the optimal policy.

Table B1 and B2 show the optimal policy in the single-gen 10 and the single-gen 30 treatment, respectively. Table B3 shows the optimal policies in multi-gen treatments. For every beginning state an optimal policy is given. Table B4 shows the optimal policies in OLG treatments. Table B5 shows the optimal policies in OLG treatments under re-optimization. The new optimal policy is calculated for every possible state with an individual in period 5.

Table B1: Optimal policy in single-gen 10 treatment with characteristic values

							$u^*(t)$ for $t \in \{0,\dots,9\}$									
$x_1(0)$	$x_2(0)$	π_0	π_μ	π_σ	π_{min}	π_{max}	0	1	2	3	4	5	6	7	8	9
0	0	5700	5826	499	5400	6600	0	0	0	0	0	0	1	1	1	2

Table B2: Optimal policy in single-gen 30 treatment with characteristic values

							$u^*(t)$ for $t \in \{0,\dots,29\}$									
$x_1(0)$	$x_2(0)$	π_0	π_μ	π_σ	π_{min}	π_{max}	0	1	...	23	24	25	26	27	28	29
0	0	14700	14826	500	14400	15700	0	0	...	0	0	0	1	1	1	2

Table B3: Optimal policy in multi-gen treatments with characteristic values

							$u^*(t)$ for $t \in \{0,\ldots,9\}$									
$x_1(0)$	$x_2(0)$	π_0	π_μ	π_σ	π_{min}	π_{max}	0	1	2	3	4	5	6	7	8	9
0	0	5700	5826	499	5400	6600	0	0	0	0	0	0	1	1	1	2
300	0	5250	5376	499	4950	6150	0	0	0	0	0	0	0	1	1	2
600	0	4800	4926	499	4500	5700	-1	0	0	0	0	0	0	1	1	2
900	0	4350	4476	499	4050	5250	-1	-1	0	0	0	0	0	1	1	2
1200	0	3900	4026	499	3600	4800	-1	-1	-1	0	0	0	0	1	1	2
600	1	4800	4926	499	4500	5700	-1	0	0	0	0	0	0	1	1	2
900	1	4350	4476	499	4050	5250	-1	-1	0	0	0	0	0	1	1	2
1200	1	3900	4026	499	3600	4800	-1	-1	-1	0	0	0	0	1	1	2
1500	1	3450	3576	499	3150	4350	-1	-1	-1	-1	0	0	0	1	1	2
1800	1	3000	3126	499	2700	3900	-1	-1	-1	-1	-1	0	0	1	1	2

Table B4: Optimal policy in OLG treatments with characteristic values

						$u^*(t)$ for $t \in \{0,\ldots,13\}$													
$x_1(0)$	$x_2(0)$	π_μ	π_σ	π_{min}	π_{max}	0	1	2	3	4	5	6	7	8	9	10	11	12	13
0	0	4525,0	459.1	3150.0	7950.0	0	0	0	1	1	1	2	2	2	2	2	2	2	2
300	0	4075.0	459.1	2700.0	7500.0	0	0	0	0	1	1	2	2	2	2	2	2	2	2
600	0	3625.0	459.1	2250.0	7050.0	-1	0	0	0	1	1	2	2	2	2	2	2	2	2
900	0	3175.0	459.1	1800.0	6600.0	-1	-1	0	0	1	1	2	2	2	2	2	2	2	2
1200	0	2725.0	459.1	1350.0	6150.0	-1	-1	-1	0	1	1	2	2	2	2	2	2	2	2
600	1	3625.0	459.1	2250.0	7050.0	-1	0	0	0	1	1	2	2	2	2	2	2	2	2
900	1	3175.0	459.1	1800.0	6600.0	-1	-1	0	0	1	1	2	2	2	2	2	2	2	2
1200	1	2725.0	459.1	1350.0	6150.0	-1	-1	-1	0	1	1	2	2	2	2	2	2	2	2
1500	1	2275.0	459.1	900.0	5700.0	-1	-1	-1	-1	1	1	2	2	2	2	2	2	2	2
1800	1	1763.4	408.9	0	5400.0	-1	-1	-1	-1	-1	1	2	2	2	2	2	2	2	2

Table B5: Optimal policy in OLG treatments with characteristic values under re-optimization during lifetime in period 5

						$u^*(t)$ for $t \in \{0,\ldots,13\}$													
$x_1(0)$	$x_2(0)$	π_μ	π_σ	π_{min}	π_{max}	0	1	2	3	4	5	6	7	8	9	10	11	12	13
0	0	2284.9	342.8	900.0	5400.0	-	-	-	-	-	1	1	2	2	2	2	2	2	2
300	0	1959.4	431.1	900.0	5400.0	-	-	-	-	-	1	1	2	2	2	2	2	2	2
600	0	1527.8	484.0	0.0	4800.0	-	-	-	-	-	1	2	2	2	2	2	2	2	2
900	0	1049.0	478.2	0.0	3600.0	-	-	-	-	-	2	2	2	2	2	2	2	2	2
1200	0	572.1	369.1	0.0	3600.0	-	-	-	-	-	2	2	2	2	2	2	2	2	2
600	1	1384.9	342.8	0.0	4500.0	-	-	-	-	-	-1	1	2	2	2	2	2	2	2
900	1	887.8	313.2	0.0	3600.0	-	-	-	-	-	-1	-1	2	2	2	2	2	2	2
1200	1	499.5	228.4	0.0	3600.0	-	-	-	-	-	-1	-1	-1	2	2	2	2	2	2
1500	1	236.9	148.1	0.0	3600.0	-	-	-	-	-	-1	-1	-1	-1	2	2	2	2	2
1800	1	90.6	82.6	0	2400.0	-	-	-	-	-	-1	-1	-1	-1	-1	2	2	2	2

To compare the single-gen 30, multi-gen, and OLG treatments, we calculate the expected payoffs over the known length of each economy (i.e., 30 periods). In the single-gen 30 treatment, this value equals the expected payoff for the single generation (i.e., 14.826 cents). For the multi-gen and OLG treatments we have to calculate the overall expected payoff as follows.

Let A^G be the set of possible age structures in the economy. Let A be a stochastic variable with realizations in A^G. We have the probability that the society has the age structure a given as $P(A = a)$. In the multi-gen treatments there is only one possible age structure and we have $A^G = A^{MG} = \{(0,1,\ldots,9,0,1,\ldots,9,0,1,\ldots,9)\}$ with probability 1. For the OLG treatments we have to determine the possible realizations to achieve the elements of the set A^{OLG}. Furthermore, we have to calculate the probability for each element to become realized. To determine the expected payoff $\pi_\mu^{sum} = \sum_{a\in A^G} P(A = a) \cdot E(\pi|a)$, we have to specify how the age structure influences the decisions of the living individuals. As done so far, each individual picks its optimal policy with the beginning of its life. So for every birth event t^* the states $x_1(t^*)$ and $x_2(t^*)$ dictate the optimal policy of the new-born individual for its entire lifetime.

The expected payoff regarding one fixed age structure a is described as $E(\pi|a)$. We calculate this value as it has already been done for one single individual. This is feasible since we can determine the optimal policies over the existence of the economy via age structure and states for every time-point. Every possible path of accumulated debt is calculated to get the expected payoff. Hence, we have 14,652 Cents expected payoff in the multi-gen treatments over the three generations. There are 1,494 different age structures in OLG treatments over the existence of the economy. This yields 13,999 Cents expected payoff accumulated over all living generations.

Pseudocode of dynamic programming

Input: x_0, N, N_x, N_u, $\tilde{X}$, $\tilde{U}$, $P(\omega_t = 1)$, $P(\nu_t = 1)$ ($\tilde{T}$ implicitly given by N)
Output: Optimal control u^*, optimal trajectory x^* (without over-indebtedness)
Allocate arrays: $J[N_x, N], x^*[n_x, N], u^*[n_u, N], xOpt[N_x, N], uOpt[N_x, N]$;
// Initialize with Mayer term
for ($i \leftarrow 1$ **to** N_x) **do** $J_{i,N} = 0$;
// Backwards loop: complete enumeration
for ($t \leftarrow N - 1$ **to** 0) **do**
  for ($i \leftarrow 1$ **to** N_x) **do**
    $P_\omega \leftarrow x_2^{(i)} + (1 - x_2^{(i)}) \cdot P(\omega_t = 1 | x^{(i)})$;
    for ($j \leftarrow 1$ **to** N_u) **do**
      Initialize $\hat{J} \leftarrow 0$;
      for ($\omega \leftarrow 1$ **to** 0) **do**
        // Calculate solution $x(t+1)$ with respect to realization ω
        $x_1(t+1) \leftarrow x_1^{(i)} + (1 - \omega) \cdot 300 \cdot u^{(j)} - \omega \cdot 300$;
        $x_2(t+1) \leftarrow \mathbb{I}_{\{>300\}}(x_1(t+1)) \cdot \omega$;
        $x_3(t+1) \leftarrow x_3^{(i)} + (1 - x_3^{(i)}) \cdot (1 - x_2(t+1)) \cdot \mathbb{I}_{\{>300\}}(x_1(t+1))$;
        Select $x^{(s)} \in \tilde{X}$, so that $x^{(s)} = x(t+1)$;
        // Calculate period pay-off
        Set $L \leftarrow L(x^{(i)}, u^{(j)}, \omega)$ as defined;
        if (*Next state* $x(t+1)$ *is feasible*) **then**
          $L \leftarrow L \cdot (1 - P(\nu_t = 1))$; // Expectation value of ν_t
          $\hat{J} \leftarrow \hat{J} + |1 - \omega - P_\omega| \cdot (L + J_{s,t+1})$; // Expectation value of ω_t
          // Select Index of next state
          if ($\omega == 1 \vee (\omega == 0 \wedge P_\omega \neq 1)$) **then** $sOpt = s$;
        end if
      end for
      if ($\hat{J} \geq J_{i,t}$) **then**
        $J_{i,t} \leftarrow \hat{J}$;
        $uOpt_{i,t} \leftarrow j$;
        $xOpt_{i,t} \leftarrow sOpt$;
      end if
    end for
  end for
end for
// Forward loop: Read solution for starting value x_0
Select $x^{(i)} \in \tilde{X}$, so that $x^{(i)} = x_0$ and set $x_0^* \leftarrow x^{(i)}$;
for ($t \leftarrow 0$ **to** $N - 1$) **do**
  $j \leftarrow uOpt_{i,t}$;
  $i \leftarrow xOpt_{i,t}$;
  $u_t^* \leftarrow u^{(j)}$;
  $x_t^* \leftarrow x^{(i)}$;
end for

References

Bellman R. The theory of dynamic programming. Bulletin of the American Mathematical Society. 1954; 60: 503-515.

Bertsekas DP. Dynamic programming and optimal control. 1st ed. Belmont, MA: Athena Scientific; 1995; 1(2).
