# Supplementary material for: The two sides of public debt: Intergenerational altruism and burden shifting
Source: PLoS One. 2018 Aug 28;13(8):e0202963. doi: 10.1371/journal.pone.0202963 (PMC6112656; doi:10.1371/journal.pone.0202963)
Supplement: S3 Appendix — (PDF) [file pone.0202963.s003.pdf]

Appendix C: Additional regressions

Appendix to section “Public debt and public good provision”

The results reported in Table 5 are analogous for the provision of public goods in the regressions 2-10a and 2-10b in Table C1. In 2-10a, we observe an overall significantly negative effect of the OLG treatment dummy on the public good provision. The combination of the significant OLG treatment dummy and its time interaction effect in regression 2-10b, however, reveals that in the early rounds, the public good provision is higher in the OLG than in the single-gen 10 treatment, but declines sharply over time. We find a similar negative time interaction effect for the multi-gen treatment dummy on the public good provision in regression 2-10b. This indicates that also in the multi-gen treatment public good provision declines over time compared to that in the single-gen 10 treatment. Note, however, that this effect is much smaller than in the OLG treatment and is not reflected in an overall effect in regression 2-10a or in any of the public debt models. The significant coefficient of the period in the regressions indicates that there is a general tendency – next to the interaction effects – for public debt to increase over time. The regression 2-30b in C1 shows that interactions do affect the public good provision in a rather complicated way, as before in the 10-period regression 2-10b. The overall treatment effects are also similar to our previous 10-period results: overall public good provision is smaller in the multi-gen and OLG treatments than in single-gen 30.

Table C1: Tobit public good regressions – Baseline treatments

	model 2-10a (10 periods)	model 2-30a (30 periods)	model 2-10b (10 periods)	model 2-30b (30 periods)
dependent variable	public good	public good	public good	public good
dummy multi-gen baseline	-31.79	-48.44***	68.38	-13.37
	(27.58)	(11.54)	(47.09)	(22.79)
dummy OLG baseline	-56.67**	-79.02***	151.00***	31.29
	(22.52)	(14.67)	(52.23)	(21.63)
Period	-0.88	-0.15	17.93***	2.87***
	(5.08)	(0.80)	(3.73)	(0.53)
period X			-18.29**	-2.26**
dummy multi-gen baseline			(8.97)	(1.10)
period X			-37.91***	-7.16***
dummy OLG baseline			(6.95)	(0.96)
Constant	347.22***	321.88***	244.34***	275.27***
	(36.06)	(12.59)	(35.22)	(7.86)
N	200	600	200	600
pseudo R squared	0.0021	0.0033	0.0102	0.0054

In models 2-10a and 2-10b only the first 10 periods are considered and the single-gen 10 treatment serves as the reference group. In models 2-30a and 2-30b, 30 periods are considered and the single-gen 30 treatment serves as the reference group. Robust standard errors are in parentheses. Test statistics: *** $p \leq 0.01$, ** $p \leq 0.05$, * $p \leq 0.1$

Appendix to section “Revealed debt preferences”

The following Table C2 displays the results of 8 Tobit regressions with robust standard errors in brackets clustered at the individual level with individual’s revealed debt preference as a dependent variable.

The first four models (models 3 to 6) relate each individual’s elicited RDP to that individual’s in-game age, elicited risk aversion measure (Holt-Laury-Task), gender, and to an indicator variable that has the value 1 if the individual’s major field of study is in economics or management. Additionally, we include the variables “lagged debt,” “last generation’s debt,” and “last period imposed tax” in the regression models. The former captures the reaction of the individual to the information on the economy’s debt in the previous period. “Last generation’s debt” captures the reaction of the individual to the information on the debt that the economy inherited from the previous generation (i.e., the debt level that each individual encountered in his very first lifetime period). “Last period imposed tax” captures the reaction of the individual to an imposed tax due to over-indebtedness in the previous period. Remember that the regression models only deal with the periods in which groups decided on the size of the public good and the debt. This excludes the periods with imposed taxes due to over-indebtedness. The indicator variable “last period imposed tax” is 1, whenever the previous period was the last period with an imposed tax.

The last four models (models 7 to 10) additionally include a squared age parameter to allow for a non-linear response of the RDP on the in-game age. Analogous regression analyses relating the individual’s proposed size of the public good and proposed tax to the discussed parameters are presented in tables C3 and C4.

Table C2: Tobit regressions – revealed debt preference (generational configurations)

	model 3 single-gen 10	model 4 single-gen 30	model 5 multi-gen baseline	model 6 OLG base-line	model 7 single-gen 10	model 8 single-gen 30	model 9 multi-gen baseline	model 10 OLG base-line
in-game age	36.06***	4.27***	30.98***	13.56***	-180.54***	-17.80***	-120.30***	13.77
	(4.84)	(0.43)	(3.21)	(4.25)	(15.49)	(1.54)	(12.44)	(15.18)
in-game age squared					18.16***	0.71***	12.56***	-0.02
					(1.43)	(0.06)	(1.19)	(1.40)
lagged debt	0.05	0.14*	0.06	0.08	-0.01	0.04	0.01	0.08
	(0.17)	(0.08)	(0.08)	(0.07)	(0.08)	(0.06)	(0.07)	(0.07)
last generation's debt			-0.02	-0.05			-0.00	-0.05
			(0.02)	(0.05)			(0.02)	(0.04)
last period imposed tax			9.67	105.06***			24.47	105.07***
			(21.02)	(27.71)			(21.93)	(27.80)
risk aversion	-5.68	0.94	3.17	-10.45	-5.62	-1.99	2.08	-10.45
	(6.07)	(5.79)	(5.92)	(9.35)	(5.68)	(5.10)	(5.93)	(9.34)
Male	36.25*	-37.57	2.37	23.12	35.22*	-47.62	1.15	23.13
	(20.01)	(41.08)	(17.60)	(25.79)	(18.43)	(38.25)	(17.54)	(25.87)
major in economics	-4.18	-37.16***	3.76	4.17	-4.19	-25.98***	6.00	4.16
	(16.29)	(11.64)	(17.90)	(26.15)	(14.43)	(7.80)	(18.18)	(26.15)
Constant	-109.68**	-1.89	-125.16***	75.19	427.64***	149.37***	269.49***	74.72
	(45.23)	(56.74)	(40.22)	(64.96)	(56.53)	(51.83)	(48.92)	(78.15)
N	153	522	550	344	153	522	550	344
pseudo R2	0.0245	0.0162	0.0180	0.0098	0.0689	0.0303	0.0353	0.0098

Robust standard errors are in parentheses. Test statistics: *** $p \leq 0.01$, ** $p \leq 0.05$, * $p \leq 0.1$

Table C3**:** Tobit regressions – public good size (generational configurations)

	model 3 single-gen 10	model 4 single-gen 30	model 5 multi-gen baseline	model 6 OLG baseline	model 7 single-gen 10	model 8 single-gen 30	model 9 multi-gen baseline	model 10 OLG baseline
in-game age	21.14***	3.35***	19.16***	19.82***	-91.60***	-12.51***	-66.59***	13.39
	(3.64)	(0.76)	(3.08)	(6.20)	(16.97)	(2.00)	(12.09)	(18.58)
in-game age squared					9.50***	0.51***	7.15***	0.59
					(1.47)	(0.06)	(1.07)	(1.87)
lagged debt	-0.11	0.02	-0.02	0.01	-0.14	-0.06	-0.06	0.01
	(0.13)	(0.06)	(0.09)	(0.09)	(0.11)	(0.05)	(0.09)	(0.09)
last generation's debt			-0.02	-0.04			-0.01	-0.04
			(0.03)	(0.07)			(0.03)	(0.07)
last period imposed tax			-21.36	73.19***			-13.45	72.99***
			(25.92)	(27.63)			(26.27)	(27.62)
risk aversion	9.55	-2.61	0.85	-15.25*	9.62	-4.71	0.26	-15.24*
	(9.79)	(4.55)	(8.23)	(8.40)	(9.80)	(4.08)	(8.14)	(8.37)
male	9.99	-6.02	39.80*	86.14***	9.81	-13.07	39.57*	85.92***
	(38.70)	(28.34)	(22.01)	(29.81)	(38.50)	(25.18)	(21.96)	(29.76)
major in economics	-23.19	-26.30**	-16.83	-8.63	-23.51	-18.27**	-15.69	-8.40
	(23.64)	(12.01)	(20.00)	(30.97)	(24.03)	(9.04)	(20.21)	(30.87)
constant	172.80***	297.83***	226.44***	304.02***	450.88***	406.24***	448.73***	318.14***
	(50.29)	(28.93)	(50.92)	(71.89)	(59.02)	(26.77)	(52.93)	(79.13)
N	153	522	550	344	153	522	550	344
pseudo R2	0.0139	0.0124	0.0111	0.0162	0.0324	0.0250	0.0184	0.0162

Robust standard errors are in parentheses. Test statistics: *** $p \leq 0.01$, ** $p \leq 0.05$, * $p \leq 0.1$

Table C4: Tobit regressions – tax (generational configurations)

	model 3 single-gen 10	model 4 single-gen 30	model 5 multi-gen baseline	model 6 OLG baseline	model 7 single-gen 10	model 8 single-gen 30	model 9 multi-gen baseline	model 10 OLG baseline
in-game age	-7.65	-2.80	-11.38***	-10.03***	76.22***	10.72**	51.54***	0.43
	(7.15)	(2.30)	(2.45)	(2.41)	(21.50)	(4.73)	(10.39)	(7.49)
in-game age squared					-6.98***	-0.42**	-5.13***	-0.95
					(2.00)	(0.20)	(0.97)	(0.69)
lagged debt	-0.11	-0.15	-0.12**	-0.07**	-0.07	-0.07	-0.10**	-0.07**
	(0.19)	(0.11)	(0.05)	(0.04)	(0.16)	(0.11)	(0.05)	(0.04)
last generation's debt			0.01	0.02			0.01	0.02
			(0.02)	(0.03)			(0.02)	(0.03)
last period imposed tax			-43.20***	-17.46			-49.79***	-17.35
			(15.69)	(13.14)			(15.93)	(13.05)
risk aversion	6.27	6.78	-5.98	10.81*	6.27	10.19	-5.33	10.86*
	(10.13)	(11.96)	(6.37)	(5.99)	(9.53)	(11.78)	(6.25)	(5.94)
male	-20.60	82.56*	17.43	-0.71	-16.30	85.02*	17.58	-0.32
	(32.11)	(46.23)	(18.04)	(11.95)	(30.57)	(46.30)	(17.72)	(11.90)
major in economics	-39.46	98.30**	-0.28	1.15	-43.01*	84.36**	-0.76	0.82
	(25.92)	(39.56)	(18.19)	(12.08)	(23.90)	(35.62)	(17.99)	(12.02)
constant	205.51**	130.46*	254.05***	98.56**	-13.79	23.34	81.64*	74.96*
	(84.00)	(73.49)	(51.05)	(38.42)	(74.71)	(59.24)	(46.77)	(40.96)
N	153	522	550	344	153	522	550	344
pseudo R2	0.0264	0.1030	0.0341	0.0270	0.0744	0.1213	0.0542	0.0278

Robust standard errors are in parentheses. Test statistics: *** $p \leq 0.01$, ** $p \leq 0.05$, * $p \leq 0.1$

Appendix to section "The effect of debt ceilings"

Table C5 displays the results of our regression analysis for the multi-gen treatments (model 11 and 12) and for the OLG treatments (model 13 and 14). As in the previous regression analysis, the coefficient of the variable "period" is positive and significant in the case of the public debt level in both settings, indicating rising public debt over time. The coefficient for the debt ceiling is non-significant in all models.

Table C5: Tobit public debt and public good regressions – Debt ceiling treatments

	model 11	model 12	model 13	model 14
	multi-gen (no OLG)		OLG	
dependent variable	public debt	public good	public debt	public good
dummy debt ceiling	32.58	-4.56	-14.35	22.54
	(29.87)	(13.66)	(30.77)	(18.37)
period	9.33***	-0.06	11.98***	-4.15***
	(2.02)	(0.95)	(1.82)	(0.85)
constant	239.47***	270.08***	268.26***	292.88***
	(38.43)	(21.27)	(30.97)	(20.44)
N	360	360	360	360
pseudo R squared	0.0095	0.0000	0.0179	0.0024

Robust standard errors are in parentheses. Test statistics: *** $p \leq 0.01$, ** $p \leq 0.05$, * $p \leq 0.1$

Table C6: Tobit regressions – revealed debt preference (debt ceiling)

	model 15<br>multi-gen	model 16<br>OLG	model 17<br>multi-gen	model 18<br>OLG
dummy debt ceiling	17.70	-4.38	21.88	-4.56
	(15.04)	(19.94)	(14.78)	(19.94)
in-game age	33.35***	17.07***	-142.04***	19.95*
	(2.47)	(3.69)	(10.25)	(11.54)
in-game age squared			14.38***	-0.27
			(0.93)	(1.13)
lagged debt	0.04	-0.07	-0.01	-0.07
	(0.07)	(0.05)	(0.06)	(0.05)
last generation's debt	-0.01	0.06	0.01	0.06
	(0.02)	(0.04)	(0.02)	(0.04)
risk aversion	5.62	-10.15	4.82	-10.17
	(4.62)	(6.27)	(4.60)	(6.27)
male	-4.49	37.07**	-4.48	37.11**
	(15.09)	(18.28)	(14.88)	(18.28)
major in economics	13.96	11.45	12.93	11.37
	(14.53)	(21.11)	(14.51)	(21.11)
constant	-146.62***	92.67*	310.58***	86.61
	(33.17)	(47.56)	(36.46)	(54.79)
N	811	704	811	704
pseudo R squared	0.0193	0.0052	0.0409	0.0052

Robust standard errors are in parentheses. Test statistics: *** $p \leq 0.01$, ** $p \leq 0.05$, * $p \leq 0.1$

Table C7: Tobit regressions – public good size (debt ceiling)

	model 15<br>multi-gen	model 16<br>OLG	model 17<br>multi-gen	model 18<br>OLG
dummy debt ceiling	10.31	1.04	13.05	1.17
	(20.77)	(21.64)	(20.84)	(21.51)
in-game age	21.19***	20.88***	-82.02***	18.58
	(2.42)	(4.23)	(9.68)	(14.55)
in-game age squared			8.50***	0.22
			(0.83)	(1.52)
lagged debt	-0.02	-0.08	-0.05	-0.08
	(0.08)	(0.05)	(0.08)	(0.05)
last generation's debt	-0.03	-0.01	-0.02	-0.01
	(0.02)	(0.05)	(0.02)	(0.05)
risk aversion	6.04	-14.21**	5.59	-14.20**
	(7.24)	(6.19)	(7.18)	(6.19)
male	32.91*	83.08***	33.19*	83.06***
	(17.94)	(19.69)	(17.80)	(19.67)
major in economics	9.38	-4.34	8.79	-4.27
	(19.55)	(25.20)	(19.62)	(25.15)
constant	185.33***	331.83***	452.70***	336.62***
	(46.49)	(50.21)	(47.93)	(54.89)
Observations	811	704	811	704
Pseudo R2	0.0124	0.0119	0.0228	0.0119

Robust standard errors are in parentheses. Test statistics: *** $p \leq 0.01$, ** $p \leq 0.05$, * $p \leq 0.1$

Table C8: Tobit regressions – tax (debt ceiling)

	model 15<br>multi-gen	model 16<br>OLG	model 17<br>multi-gen	model 18<br>OLG
dummy debt ceiling	8.59	11.73	7.19	11.43
	(14.29)	(9.37)	(14.40)	(9.37)
in-game age	-11.57***	-7.93***	60.68***	-2.79
	(2.07)	(1.96)	(9.33)	(6.22)
in-game age squared			-5.82***	-0.48
			(0.85)	(0.63)
lagged debt	-0.14***	-0.03	-0.11**	-0.03
	(0.05)	(0.02)	(0.05)	(0.02)
last generation's debt	0.00	-0.01	-0.01	-0.01
	(0.02)	(0.02)	(0.02)	(0.02)
risk aversion	-9.24*	8.29**	-8.65*	8.29**
	(5.08)	(3.47)	(5.00)	(3.46)
male	21.59	3.41	21.34	3.51
	(15.12)	(8.79)	(14.83)	(8.79)
major in economics	-7.66	1.86	-6.97	1.69
	(14.47)	(10.25)	(14.40)	(10.23)
constant	281.63***	88.56***	83.49**	77.58***
	(44.36)	(25.59)	(35.81)	(27.16)
Observations	811	704	811	704
Pseudo R2	0.0339	0.0147	0.0572	0.0149

Robust standard errors are in parentheses. Test statistics: *** $p \leq 0.01$, ** $p \leq 0.05$, * $p \leq 0.1$
