# Supplementary material for: The two sides of public debt: Intergenerational altruism and burden shifting
Source: PLoS One. 2018 Aug 28;13(8):e0202963. doi: 10.1371/journal.pone.0202963 (PMC6112656; doi:10.1371/journal.pone.0202963)
Supplement: S4 Appendix — (PDF) [file pone.0202963.s004.pdf]

Appendix D: Robustness checks

D1: Robustness check 1: Unanimity

In the following, we analyze how robust these results are with respect to the voting mechanism. To test the robustness of our results with a stricter social choice mechanism, we conducted an additional treatment in which removing and installing the debt ceiling requires unanimity in the popular vote instead of the simple majority. Hence, the debt ceiling in this treatment is only removed (or reinstalled) if all group members agree. From the literature we know that the voting mechanism used in a group decision process may indeed have an influence on the group decisions. Gillet et al. (2009) show in a common pool resource experiment with groups that groups show more efficient behavior than individuals if they have to use the unanimity rule. Under majority voting this was not the case.

The main results are presented in Tables D1 and D2, as well as in Figures D1 and D2. Surprisingly, we do not observe any significant differences between the decision patterns in the unanimity treatments and our previous majority treatments (Mann-Whitney U-test, p-values above 0.1, two-tailed). As in the previous sections, we ran Tobit regressions with public debt and public good size as dependent variables (see Table D3). We find no significant influence of this unanimity treatment variable in any of the regressions. Therefore, we conclude that even in an environment in which the debt ceiling can only be removed if all group members agree, we observe no deceleration of debt accumulation.

Table D1: Average observed parameters – Majority and unanimity treatments

treatment	# of independent observations (# of subjects)	average public good provision	average public debt	average number of periods with over-indebtedness	average number of periods with imposed tax
multi-gen DC (majority)	4 (36)	277	417	4.00	6.25
multi-gen DC (unanimity)	4 (36)	289	389	3.00	4.50
OLG DC (majority)	6 (75)	271	440	4.50	6.33
OLG DC (unanimity)	6 (72)	261	460	4.17	6.83

Table D2: Deficit behavior (unanimity)

treatment	deficit		balanced budget		voluntary surplus		imposed austerity	
	rel. freq	mean	rel. freq	mean	rel. freq	mean	rel. freq	mean
multi-gen DC (majority)	0.43	189.7	0.32	0	0.05	95.0	0.21	237.0
multi-gen DC (unanimity)	0.39	167.9	0.38	0	0.08	63.3	0.15	223.3
OLG DC (majority)	0.49	174.8	0.24	0	0.06	132.0	0.21	247.5
OLG DC (unanimity)	0.41	194.0	0.33	0	0.03	45	0.23	212.2

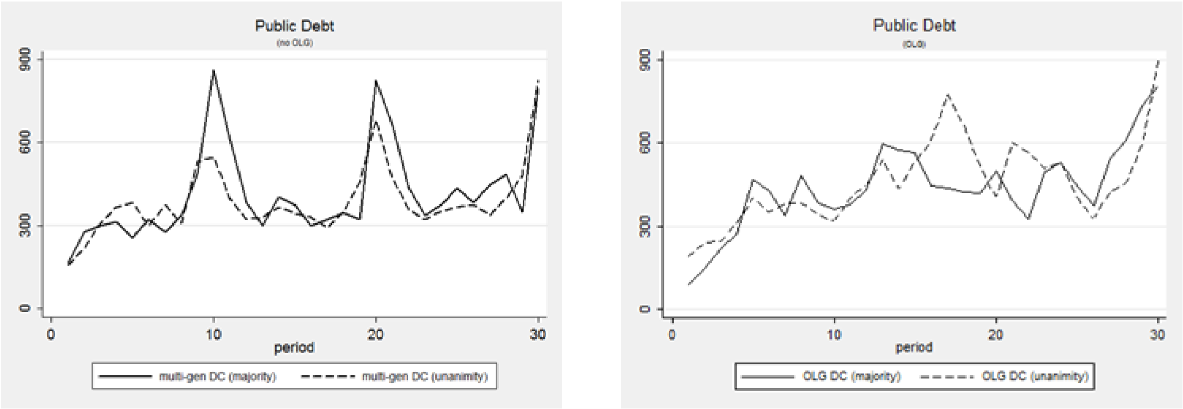

Figure D1: Public debt level with majority and unanimity voting on debt ceiling (no OLG and OLG)

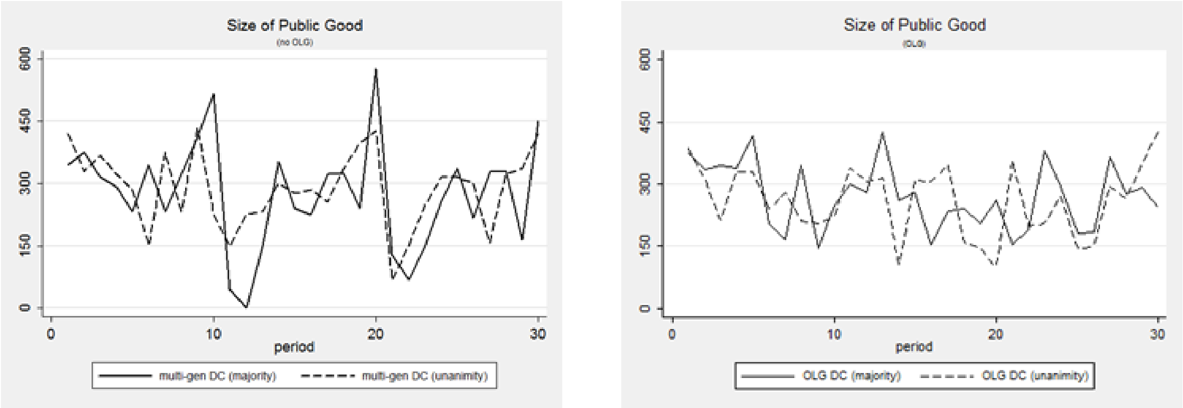

Figure D2: Size of Public Good with majority and unanimity voting on debt ceiling (no OLG and OLG)

Table D3: Tobit regressions – unanimity

	model 19	model 20	model 21	model 22
	multi-gen (no OLG)		OLG	
dependent variable	public debt level	size of public good	public debt level	size of public good
dummy unanimity	-27.88	16.47	5.20	9.41
	(22.03)	(10.95)	(38.92)	(22.85)
Period	6.91***	-1.26	11.58***	-3.30***
	(1.76)	(1.20)	(1.84)	(1.13)
Constant	309.54***	282.53***	274.46***	280.35***
	(38.91)	(20.97)	(31.32)	(22.78)
N	240	240	360	360
pseudo R squared	0.0074	0.0004	0.0150	0.0015

Robust standard errors are in parentheses. Test statistics: *** $p \leq 0.01$, ** $p \leq 0.05$, * $p \leq 0.1$

D2: Robustness check 2: OLG with friends

One limitation of our design is that bequest motives only play a minor role in our treatments with anonymous strangers. To test whether bequest motives influence behavior when there are social ties across generations, we ran an additional treatment as a robustness check. For this purpose, we used the design of the OLG baseline treatment, but invited groups of friends as our subjects. The only criterion to participate in the experiment as a group of friends is that all individuals must have been friends for at least one year.

For each economy, we recruited 3 groups of 5 friends, one for each possible generation. Over time, we replaced each member of the economy whose lifetime ended with one of his or her friends. Note that the three individuals active in an economy were never from the same group of friends.

The main results of this treatment compared to the results of the OLG baseline treatment are shown in Tables D4 and D5, as well as in figure D3. Again, we do not observe any different decision patterns in the OLG treatment with friends compared to the treatment with strangers. None of the comparisons between the main variables are significant (Mann-Whitney U-test, p-values above 0.1, two-tailed).

As in the previous sections, we ran Tobit regressions with public debt and public good size as dependent variables (see Table D6). To control for bequest motives, we use a dummy variable “friends”. In both regression analyses, we find no significant influence of this dummy variable. Therefore, we can conclude that even in an environment with friends in which intergenerational ties may evoke bequest motives, debt accumulation strongly harming future generations is not reduced.

Table D4: Average observed parameters – Friends treatment

treatment	independent observations (number of subjects)	average public good provision	average public debt	average number of periods with over-in-debtedness	average number of periods with imposed tax
OLG baseline	6 (74)	256	455	4.67	7.33
OLG baseline with friends	6 (73)	237	454	4.33	7.67

Table D5: Deficit behavior (friends)

treatment	deficit		balanced budget		voluntary surplus		imposed austerity	
	rel. freq	mean	rel. freq	mean	rel. freq	mean	rel. freq	mean
OLG baseline	0.52	158.1	0.17	0	0.07	57.5	0.24	223.0
OLG baseline with friends	0.48	165.5	0.17	0	0.09	91.9	0.26	214.6

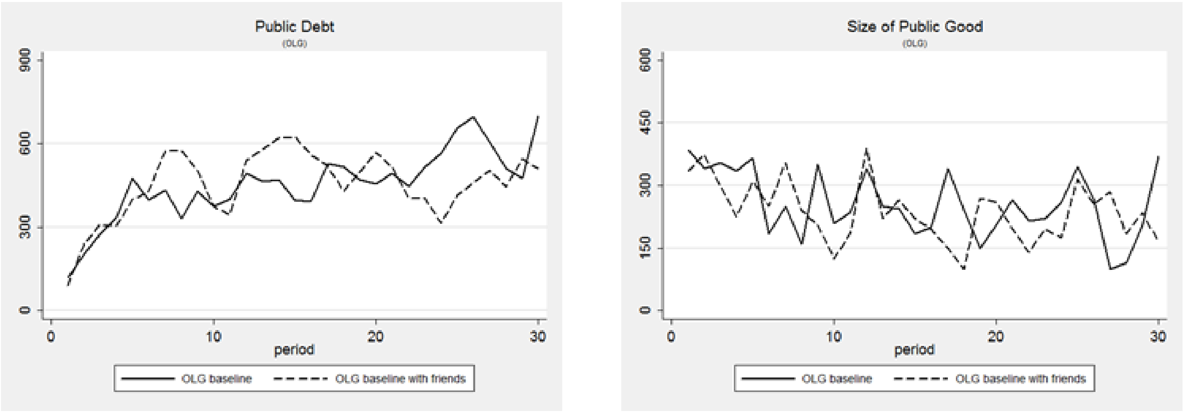

Figure D3: Public debt level and public good size with and without friends

Table D6: Tobit regressions – friends

	model 23	model 24
dependent variable	public debt level	size of public good
dummy friends	-0.67	-18.74
	(38.93)	(18.59)
Period	8.60***	-4.82***
	(2.21)	(0.75)
Constant	320.63***	302.60***
	(36.53)	(19.95)
N	360	360
pseudo R squared	0.0084	0.0031

Robust standard errors are in parentheses. Test statistics: *** $p \leq 0.01$, ** $p \leq 0.05$, * $p \leq 0.1$

References

Gillet J, Schram A, Sonnemans J. The tragedy of the commons revisited: The importance of group decision-making. Journal of Public Economics. 2009; 93(5-6): 785-797.
