# Supplementary material for: The two sides of public debt: Intergenerational altruism and burden shifting
Source: PLoS One. 2018 Aug 28;13(8):e0202963. doi: 10.1371/journal.pone.0202963 (PMC6112656; doi:10.1371/journal.pone.0202963)
Supplement: S1 File — (PDF) [file pone.0202963.s005.pdf]

Instructions

The experiment that you will participate in consists of two parts, in which you have the possibility to earn money. Before each part of the experiment, you will receive instructions explaining that part of the experiment. Next, that part of the experiment starts. After the second part of the experiment, the experiment ends and you will receive a payment that depends on the results of both parts of the experiment.

Please note that you are neither allowed to communicate with other participants nor allowed to leave your desk during the entire experiment. Please read the instructions thoroughly. If you have any questions, please, raise your hand. We will then come to you to answer your questions.

You will now receive the instructions for the first part of the experiment

.

Desk Number:

Instructions for Part 1 of the Experiment (Please fill in your desk number on the right hand side above)

Please choose one of the two lotteries A or B in each of the following 10 decision situations by marking the corresponding box in the table.

You will make a decision for all 10 situations, but your payoff from this part of the experiment is determined only by the one situation that is randomly drawn after the experiment.

In each situation, you can either earn 2,00 € or 1,60 € from lottery A and either 3,85 € or 0,10 € from lottery B. The probabilities of winning, however, vary from situation to situation. The further down you move in the table, the higher is the probability of the higher payment and the lower is the probability of the lower payment.

After part 1 and 2 of the experiment are completed, you will be asked to roll a ten-faced die two times. Once to determine one of the ten decision situations and a second time to determine your payoff from the lottery that you have chosen in this decision situation. If the number you roll is lower or equals the probability of the higher payoff, you will receive the higher payoff. Otherwise, you will receive the lower payoff. Please note that the "1" on the die corresponds to the "10%" in the table; the "2" to the "20%" and so forth up to the "9." The "0" corresponds to the "100%."

	Lottery A	Lottery B	Your decision	
			A	B
1.	2,00 € with 10% or 1,60 € with 90%	3,85 € with 10% or 0,10 € with 90%	o	o
2.	2,00 € with 20% or 1,60 € with 80%	3,85 € with 20% or 0,10 € with 80%	o	o
3.	2,00 € with 30% or 1,60 € with 70%	3,85 € with 30% or 0,10 € with 70%	o	o
4.	2,00 € with 40% or 1,60 € with 60%	3,85 € with 40% or 0,10 € with 60%	o	o
5.	2,00 € with 50% or 1,60 € with 50%	3,85 € with 50% or 0,10 € with 50%	o	o
6.	2,00 € with 60% or 1,60 € with 40%	3,85 € with 60% or 0,10 € with 40%	o	o
7.	2,00 € with 70% or 1,60 € with 30%	3,85 € with 70% or 0,10 € with 30%	o	o
8.	2,00 € with 80% or 1,60 € with 20%	3,85 € with 80% or 0,10 € with 20%	o	o
9.	2,00 € with 90% or 1,60 € with 10%	3,85 € with 90% or 0,10 € with 10%	o	o
10.	2,00 € with 100% or 1,60 € with 0%	3,85 € with 100% or 0,10 € with 0%	o	o

Instructions for Part 2 of the Experiment

single-gen treatment:

You are randomly assigned to two other participants with whom you matched in a group of three that remains together during the entire part 2 of the experiment. The part 2 of the experiment has 30 rounds.

multi-gen treatments:

You are randomly assigned to two other participants with whom you matched in a group of three that remains together during the entire part 2 of the experiment. Three such groups of three take part in the experiment, each ***successively*** making their decisions for 10 rounds. The decisions of each group of three affect the following group(s) of three.

Your group of three is second to make its decisions, i.e. in the rounds 11-20. So there was a group of three before your group of three (rounds 1-10) and another group of three follows in the rounds 21-30, faced with the same decisions as your group of three.

OLG treatments:

In this experiment, you will be part of a group consisting of exactly three members. This group of three makes decisions over 30 rounds. While the group always consists of three members, it will not have an identical constellation in all 30 rounds. Each member of the group of three exits the experiment with a certain probability after each round. The exit probability depends on the number of rounds that the group member has already participated in the experiment and increases with the number of rounds (see table below).

**individual round of the group member**	**probability of the group member exiting the experiment after this round**
1	0%
2	0%
3	0%
4	0%
5	10%
6	20%
7	30%
8	40%
9	50%
10	60%
11	70%
12	80%
13	90%
14	100%

This means:

- Since the probability of exiting the experiment in the first four rounds is 0%, every group member participates in the experiment for at least five rounds.
- If a group member has participated in the experiment for 14 rounds, he or she will definitely exit the experiment after the 14th round.

Please note:

- After every single round, a random draw determines for each current member of the group of three, whether he or she will exit the experiment or not.
- The experiment has a total of exactly 30 rounds. Hence, the experiment definitely terminates after the 30th round.

For each member of the group that exits another person is assigned to the group, so that the group again consists of three members. The exiting member then receives his or her payoff from part 1 and 2 of the experiment in cash and has to leave the laboratory.

> **OLG (baseline, DC (majority), DC (unanimity)):** The participants with the desk numbers 1, 2, and 3 are the first members of the group of three. Once the random draw determines that one of the members has to exit the experiment, the participant with the desk number 4 replaces this member. When another member exits the experiment, it is the turn of the participant with the desk number 5. And so on.
>
> **OLG (baseline with friends):** The 3 participants with the number 1 from the orange, green, and blue team are first members of the group of three. Once the random draw determines that one of the members has to exit the experiment, the participant with the number 2 from the same team replaces this member. If the experiment is also over for this participant, it is the turn of the participant with the number 3 from the *same* team. And so on. Please note: If one member of the group of three exists the experiment, he or she is always replaced by person from the same team (orange, green, blue). Hence, the group of three always consists of a member from the orange, the green, and the blue team.

In case there are more individuals present than can participate in the experiment, those individuals who were not assigned to the group of three will receive 10 Euro for part 2 of the experiment.

Your task in the experiment

In each round, every member of your group of three receives an endowment of 100 cents. Your group has to make two decisions in each round.

1. Your group has to decide whether or not to create a public facility and what size it should have if created. For every cent invested in this facility, each of the three group members receives a payoff of 0.5 cents.

2. Your group has to decide how the public facility is funded. Two funding sources are available: Taxes paid from the endowment or loans can be taken out.

Decision process

all treatments, but not treatments with DC (unanimity): Your group decides by popular vote. All votes implement the median voter principle. Hence, the median proposal is always chosen from the proposals of the three group members.

treatments with DC (unanimity): Your group decides by popular vote. When voting on the size of the public facility and on its funding, the median voter principle is implemented. Hence, the median proposal is always chosen from the proposals of the three group members.

Examples:

	Example 1	Example 2	Example 3	Example 4
group member 1	210	570	150	480
group member 2	450	570	390	480
group member 3	270	420	150	480
voting decision	270	570	150	480

Decision on the size of the public facility

With the first decision your group decides on the size of the public facility. For this purpose, each group member makes a personal proposal. The following proposal for the size of the public facility can be made: 0, 30, 60, 90, 120, 150, 180, 210, 240, 270, 300, 330, 360, 390, 420, 450, 480, 510, 540, 570, and 600 cents. The median proposal of the three group members determines the size of the public facility.

In each round, there is a new vote on the size of the public facility. Hence, the size of the public facility may vary from round to round.

Decision on the financing of the public facility

The public facility can be funded with *taxes* and/or with *loans taken out by the group*. If collected, taxes are paid from each group member's endowment (per capita tax).

If the total tax revenue (sum of the per capita tax that you and the other two group members have paid) is greater than the amount that is needed to finance the public facility, a deposit is credited to the group's account. This deposit can only be used to finance the public facility in a later round. A direct payoff of a deposit to the group members will not take place.

If the total tax revenue is not sufficient to finance the chosen size of the public facility, the missing funds will be covered by a loan to your group. Your group can voluntarily repay its debt (i.e. its outstanding loans) in future rounds by creating a deposit (i.e. if the total tax revenue is greater than the amount that is needed to finance the public facility). However,

your group is only obliged to repay the debt, if you group is overindebted. When this is the case and how repayments are then made, is explained below.

single-gen treatment: Your group's deposits or debts are registered on a separate "group account." Before each decision, the balance of this group account is shown to you and the other two group members. If the balance of your group account is positive, your group has a deposit. If the balance of your group account is negative, your group is in debt. The balance of the group account at the end of a round is transferred to the next round.

multi-gen treatments: Your group's deposits or debts are registered on a separate "group account." Before each decision, the balance of this group account is shown to you and the other two group members. If the balance of your group account is positive, your group has a deposit. If the balance of your group account is negative, your group is in debt. The balance of the group account at the end of a round is transferred to the next round. Please note that the balance of the group account at the end of the last round of one group of three is the balance of the group account at the beginning of the first round for the next group of three.

OLG treatments: Your group's deposits or debts are registered on a separate "group account." Before each decision, the balance of this group account is shown to you and the other two group members. If the balance of your group account is positive, your group has a deposit. If the balance of your group account is negative, your group is in debt. The balance of the group account at the end of a round is transferred to the next round. Please note that the balance of the group account is carried over continuously even after a member of the group of three exits the experiment and is replaced by a new person after the round.

After all group members are informed on the chosen size of the public facility, the group votes on the size of the per capita tax in round. To this end, each group member makes an individual proposal. Feasible proposals are all integers between 0 to 100 cents, as well as the numbers 0 and 100 that can also be chosen. Just as in the vote on the size of the public facility, the median proposal of the three group members determines the size of the per capita tax. **Every member of the group pays the same tax.**

In each round, there is a new vote on the size of the per capita tax.

Round payoff

Your round payoff is determined by the following formula:

$$\text{round payoff} = \underbrace{100}_{\text{endowment}} - \text{per capita tax} + 0.5 \cdot \text{size of the public facility}$$

Please note that the balance of the group account does not directly influence your payoff in the experiment. Hence, neither a positive balance (deposit) nor a negative balance (debt) on your group account will increase or decrease your payoff at the end of the experiment.

Examples

	Example 1	Example 2	Example 3
<u>decision on the size of the public facility</u>			
suggestion group member 1	210	570	150
suggestion group member 2	450	570	390
suggestion group member 3	270	420	150
➔ **chosen size**	**270**	**570**	**150**
<u>decision on the funding</u>			
suggestion group member 1	45	10	20
suggestion group member 2	60	5	80
suggestion group member 3	75	25	70
➔ **chosen per capita tax**	**60**	**10**	**70**
**total tax revenue** (= 3 · per capita tax)	**180**	**30**	**210**
**change in group account balance** (= total tax revenue – size)	**–90**	**–540**	**+60**
**round payoff per group member** (= 100 – per capita tax + 0,5 · size)	**175**	**375**	**105**

<u>treatments without DC:</u>

[no further text]

<u>treatments with DC:</u>

Debt ceiling

A debt ceiling is installed at a debt level of 300 cents (i.e. the group account balance is -300 cents). This debt ceiling prevents your group from surpassing 300 cents of debts. While the debt ceiling is installed by default at the outset of the experiment, your group does have the possibility to abolish the debt ceiling in every round. If the debt ceiling is abolished, a debt level of more than 300 cents is possible.

> **<u>DC (majority) treatments:</u>** At the beginning of every round the group decides whether to abolish the debt ceiling or not. To do so, each group member votes for one of the two proposals: “abolish the debt ceiling” or “keep the debt ceiling.” The proposal with the majority of the votes is chosen.
>
> After the debt ceiling is abolished in a round, it can be reinstalled in a later round. This is again implemented by a voting at the beginning of every round. Each group member can either vote for the proposal “reinstall the debt ceiling” or “keep the debt ceiling

abolished." If the group decides to reinstall the debt ceiling, it can be abolished again in a later round.

DC (unanimity) treatments: At the beginning of every round the group decides whether to abolish the debt ceiling or not. To do so, each group member votes for one of the two proposals: "abolish the debt ceiling" or "keep the debt ceiling." If *all three* group members vote to abolish the debt ceiling, it is abolished. If *not all three* group members vote to abolish the debt ceiling, it will remain installed. In contrast to the decisions on the size of the public facility and the per capita tax, this decision is not based on the median voter principle, but on the *unanimity rule*.

After the debt ceiling is abolished in a round, it can be reinstalled in a later round. This is again implemented by a voting at the beginning of every round. Each group member can either vote for the proposal "reinstall the debt ceiling" or "keep the debt ceiling abolished." If the group decides to reinstall the debt ceiling, it can be abolished again in a later round. Here again the unanimity rule is implemented. Hence, if *all three* group members vote to reinstall the debt ceiling, it is reinstalled. If *not all three* group members vote to reinstall the debt ceiling, it remains abolished. If the group decides to reinstall the debt ceiling, it can be abolished again in a later round.

multi-gen treatments: Please note: The decision of a group of three to abolish or to reinstall the debt ceiling also has an effect on the following group(s) of three. If, for example, the first group of three decides to abolish the debt ceiling and does not reinstall it, the debt ceiling will not be installed in the first round of the second group of three. Whether the debt ceiling is installed or not, is displayed at the beginning of each round.

OLG treatments: Please note: The decision of a group of three to abolish or to reinstall the debt ceiling also has an effect on the following group members. If, for example, a group of three decides to abolish the debt ceiling and one of the group members exits the experiment after the round, the debt ceiling will not be installed at the outset of the next round, in which a new group member replaces the group member, who exited the experiment. Whether the debt ceiling is installed or not, is displayed at the beginning of each round.

If the debt ceiling is installed, the group of three can only use the total tax revenue from the per capita tax and the maximum debt of 300 cents to finance the public facility. Has the debt level reached 300 cents, the public facility is *solely* financed by the per capita tax. Hence, in that case, the size of the public facility can at most be 300 cents per round. Examples:

	Example 1	Example 2	Example 3
**(1) group account balance at beginning of round**	**0**	**-180**	**-300**
(2) possible debt<br>(= 300 + balance of group account)	300	120	0
(3) maximum total tax revenue	300	300	300

**(4) maximum size of public facility** (=(2) + (3), but maximum 600)	600	420	300

Please note that when a debt ceiling is installed, the per capita tax must be chosen to cover the financing of the public facility. Hence, in example 2, a public facility of 420 cents can only be implemented by paying a per capita tax of 100 cents. For a public facility of 390 cents the per capita tax must at least be set at 90 cents. If your group of three decides on a per capita tax that is too low, you will receive a corresponding error message and will not reach the next round, but instead, you will have to vote again on the per capita tax.

Overindebtedness

treatments without DC: If your group has more than 300 cents in debt (i.e. the balance of the group account is smaller than -300 cents), then it is possible that your group is overindebted. Whether your group is overindebted is determined by a random draw at the end of each round. The probability for overindeptedness depends on your group's debt in the current round and is higher the larger the debt is (see the table below). The result of the random draw is displayed to you at the end of each round.

treatments with DC: If the debt ceiling is disabled, your group can make more than 300 cents in debts. If your group has more than 300 cents in debt (i.e. the balance of the group account is smaller than -300 cents), then it is possible that your group is overindebted. Whether your group is overindebted is determined by a random draw at the end of each round. The probability for overindeptedness depends on the your group's debt in the current round and is higher the larger the debt is (see the table below). The result of the random draw is displayed to you at the end of each round.

group account balance	probability of overindebtedness
not smaller than -300	0%
-400 to -301	10%
-500 to -401	20%
-600 to -501	30%
-700 to -601	40%
-800 to -701	50%
-900 to -801	60%
-1000 to -901	70%
-1100 to -1001	80%
-1200 to -1101	90%
less than -1.200	100%

This means:

- If your group has no more than 300 cents debt, your group is definitely not overindebted.
- If your group has more than 1200 cents debt, your group is definitely overindebted.
- If your group, for example, has 840 cents debt, then a random draw with a probability of 60% will determine whether your group is overindebted.

single-gen treatment: *If your group is overindebted in a round, a compulsory tax is charged in the **following round***. Please note that this means that the overindebtedness only influences future payoffs and not the payoff of the current round.

multi-gen treatment: *If your group is overindebted in a round, a compulsory tax is charged in the **following round***. Please note that this means that the overindebtedness only influences future payoffs and not the payoff of the current round. Please also note that an overindebtedness in the last round of your group of three leads to a compulsory taxation of the following group of three.

OLG treatments: *If your group is overindebted in a round, a compulsory tax is charged in the **following round***. Please note that this means that the overindebtedness only influences future payoffs and not the payoff of the current round. Please also note that if a group member exits the experiment, the new member, who replaces him or her, has to pay the compulsory tax.

The compulsory tax, paid from the endowment by each group member, is 100 cents at maximum for each group member and is charged until your group's debt is decreased to 300 cents. The computer automatically determines the compulsory tax to decrease the debt as fast as possible to this level.

Examples for the calculation of the compulsory tax:

	Example 1	Example 2
group account balance at the beginning of round	-570	-960
necessary total repayment due to overindebtedness	270	660
compulsory tax per capita (maximum 100)	90	100
total sum of the compulsory tax payments	270	300
group account balance at end of round	-300	-660

single-gen treatment: If the total sum of the compulsory tax payments of all group members in one round is insufficient to decrease the debt to 300 cents (as in example 2), a compulsory tax will be automatically charged in the following round or, if necessary, in a number of following rounds.

multi-gen treatments: If the total sum of the compulsory tax payments of all group members in one round is insufficient to decrease the debt to 300 cents (as in example 2), a compulsory tax will be automatically charged in the following round or, if necessary, in a number of following rounds. Please note that the repeated collection of the compulsory tax is also possible in the following group of three, if the group account balance in the last round of your group of three still has more than 300 cents in debt despite the compulsory taxation.

OLG treatments: If the total sum of the compulsory tax payments of all group members in one round is insufficient to decrease the debt to 300 cents (as in example 2), a compulsory tax will be automatically charged in the following round or, if necessary, in a number of following rounds. Please note that if a group member exits the experiment, the repeated collection of the compulsory tax may also affect the new member or possibly the new members, who replace him or her.

If a compulsory tax is charged in a round, no public facility is available in that round. In this case, your round payoff is determined by:

$$\text{Payoff per round} = \underbrace{100}_{\text{endowment}} - \text{compulsory tax}$$

treatments without DC: [no further text]

treatments with DC: Furthermore, there is no vote on the debt ceiling in a round, in which a compulsory tax is charged.

As soon as your group's debt is decreased to 300 cents after a period of overindebtedness, a public facility will be available in the following round, the size and financing of which is decided by your group.

single-gen treatment: After you and your group have completed the 30 rounds of part 2 of the experiment, you will be paid the total sum of your round payoffs in cash.

multi-gen treatments: After you and your group have completed the 10 rounds of part 2 of the experiment, you will be paid the total sum of your round payoffs in cash.

OLG treatments: After you have completed part 2 of the experiment, you will be paid the total sum of your round payoffs in cash.

Before the part 2 of the experiment starts, you are asked to answer some questions at your computer. Answering these questions only serves as a test of your comprehension and is not relevant for your payoff.

single-gen treatment:

Subsequently, 10 practice rounds are conducted, in which you make the above mentioned decisions in a group of three. The assignment of participants to the groups of three is carried out randomly. However, we make sure that you will not be in a group with the same members in the actual experiment. The practice rounds are meant for you to become acquainted with the computer program and are therefore not relevant to your payoff.

After completing these 10 practice rounds, you will start with the part 2 of the experiment that is relevant to your payoff.

multi-gen treatments:

Subsequently, 2 trial runs are conducted (one trial run with 10 practice rounds and one trial run with 5 practice rounds), in which you make the above mentioned decisions in a group of three. The assignment of participants to the groups of three is carried out randomly. However, we make sure that you will not be in a group with the same members in the actual experiment. The trial runs are meant for you to become acquainted with the computer program and are therefore not relevant to your payoff.

> **multi-gen (baseline):** In the first trial run (total of 10 practice rounds), it is assumed that the decisions of the group of three preceding your group of three led to a group account balance of 0 cents after their 10th round. Hence, your group's account balance at the beginning of your first practice round is 0 cents.
>
> In the second trial run (total of 5 practice rounds), it is assumed that the decisions of the group of three preceding your group of three led to a group account balance of -600 cents after their 10th round. Hence, your group's account balance at the beginning of your first practice round is -600 cents.
>
> **multi-gen (DC (majority), DC (unanimity)):** In the first trial run (total of 10 practice rounds), it is assumed that the group of three preceding your group of three (i.e. in the rounds 1-10) has not abolished the debt ceiling and that their decisions led to a group account balance of 0 cents after their 10th round. Hence, your group's account balance at the beginning of your first practice round is 0 cents.
>
> In the second trial run (total of 5 practice rounds), it is assumed that the group of three preceding your group of three has abolished the debt ceiling and that their decisions led to a group account balance of -600 cents after their 10th round. Hence, your group's account balance at the beginning of your first practice round is -600 cents. It is also assumed that there has been no overindebtedness yet.

After the first trial run is completed, the second trial run starts. Subsequently, you are led to the room next door, where the part 2 of the experiment is conducted that is relevant to your payoff.

OLG treatments:

Subsequently, 2 trial runs are conducted (one trial run with 10 practice rounds and one trial run with 5 practice rounds), in which you make the above mentioned decisions in a group of three. The assignment of participants to the groups of three is carried out randomly. However, we make sure that you will not be in a group with the same members in the actual experiment. Please note that it is not possible to be eliminated from the experiment in the trial runs. The trial runs are meant for you to become acquainted with the computer program and are therefore not relevant to your payoff.

> **OLG (baseline, baseline with friends):** In the first trial run (total of 10 practice rounds), it is assumed that your group's account balance at the beginning of your first practice round is 0 cents.
>
> In the second trial run (total of 5 practice rounds), it is assumed that your group's account balance at the beginning of your first practice round is -600 cents. However, overindebtedness has not occurred yet.
>
> **OLG (DC (majority), DC (unanimity)):** In the first trial run (total of 10 practice rounds), it is assumed that the debt ceiling has not been abolished and that your group's account balance at the beginning of your first practice round is 0 cents.
>
> In the second trial run (total of 5 practice rounds), it is assumed that the debt ceiling has been abolished and that your group's account balance at the beginning of your first practice round is -600 cents. However, overindebtedness has not occurred yet.
>
> **OLG (baseline, DC (majority), DC (unanimity)):** After the first trial run is completed, the second trial run starts. Subsequently, the persons with the desk numbers 1, 2, and 3 are led to the room next door, where the part 2 of the experiment is conducted that is relevant to your payoff.
>
> **OLG (baseline with friends):** After the first trial run is completed, the second trial run starts. Subsequently, the 3 persons with the desk number 1 from the orange, the green, and the blue team are led to the room next door, where the part 2 of the experiment is conducted that is relevant to your payoff.

The table below displays your round payoffs and the changes in your group’s account balance, when the size of the public facility and the per capita tax are varied.

per capita tax	total tax revenue	size: 0		size: 30		size: 60		size: 90		size: 120		size: 150		size: 180		size: 210		size: 240		size: 270		size: 300	
**0**	**0**	100	0	115	-30	130	-60	145	-90	160	-120	175	-150	190	-180	205	-210	220	-240	235	-270	250	-300
**10**	**30**	90	30	105	0	120	-30	135	-60	150	-90	165	-120	180	-150	195	-180	210	-210	225	-240	240	-270
**20**	**60**	80	60	95	30	110	0	125	-30	140	-60	155	-90	170	-120	185	-150	200	-180	215	-210	230	-240
**30**	**90**	70	90	85	60	100	30	115	0	130	-30	145	-60	160	-90	175	-120	190	-150	205	-180	220	-210
**40**	**120**	60	120	75	90	90	60	105	30	120	0	135	-30	150	-60	165	-90	180	-120	195	-150	210	-180
**50**	**150**	50	150	65	120	80	90	95	60	110	30	125	0	140	-30	155	-60	170	-90	185	-120	200	-150
**60**	**180**	40	180	55	150	70	120	85	90	100	60	115	30	130	0	145	-30	160	-60	175	-90	190	-120
**70**	**210**	30	210	45	180	60	150	75	120	90	90	105	60	120	30	135	0	150	-30	165	-60	180	-90
**80**	**240**	20	240	35	210	50	180	65	150	80	120	95	90	110	60	125	30	140	0	155	-30	170	-60
**90**	**270**	10	270	25	240	40	210	55	180	70	150	85	120	100	90	115	60	130	30	145	0	160	-30
**100**	**300**	0	300	15	270	30	240	45	210	60	180	75	150	90	120	105	90	120	60	135	30	150	0

per capita tax	total tax revenue	size: 330		size: 360		size: 390		size: 420		size: 450		size: 480		size: 510		size: 540		size: 570		size: 600	
**0**	**0**	265	-330	280	-360	295	-390	310	-420	325	-450	340	-480	355	-510	370	-540	385	-570	400	-600
**10**	**30**	255	-300	270	-330	285	-360	300	-390	315	-420	330	-450	345	-480	360	-510	375	-540	390	-570
**20**	**60**	245	-270	260	-300	275	-330	290	-360	305	-390	320	-420	335	-450	350	-480	365	-510	380	-540
**30**	**90**	235	-240	250	-270	265	-300	280	-330	295	-360	310	-390	325	-420	340	-450	355	-480	370	-510
**40**	**120**	225	-210	240	-240	255	-270	270	-300	285	-330	300	-360	315	-390	330	-420	345	-450	360	-480
**50**	**150**	215	-180	230	-210	245	-240	260	-270	275	-300	290	-330	305	-360	320	-390	335	-420	350	-450
**60**	**180**	205	-150	220	-180	235	-210	250	-240	265	-270	280	-300	295	-330	310	-360	325	-390	340	-420
**70**	**210**	195	-120	210	-150	225	-180	240	-210	255	-240	270	-270	285	-300	300	-330	315	-360	330	-390
**80**	**240**	185	-90	200	-120	215	-150	230	-180	245	-210	260	-240	275	-270	290	-300	305	-330	320	-360
**90**	**270**	175	-60	190	-90	205	-120	220	-150	235	-180	250	-210	265	-240	280	-270	295	-300	310	-330
**100**	**300**	165	-30	180	-60	195	-90	210	-120	225	-150	240	-180	255	-210	270	-240	285	-270	300	-300
